# Multilocus Sequence Analysis of Nectar Pseudomonads Reveals High Genetic Diversity and Contrasting Recombination Patterns

**DOI:** 10.1371/journal.pone.0075797

**Published:** 2013-10-08

**Authors:** Sergio Álvarez-Pérez, Clara de Vega, Carlos M. Herrera

**Affiliations:** Estación Biológica de Doñana, Consejo Superior de Investigaciones Científicas (CSIC), Sevilla, Spain; Centro de Investigación y de Estudios Avanzados, Mexico

## Abstract

The genetic and evolutionary relationships among floral nectar-dwelling *Pseudomonas* ‘sensu stricto’ isolates associated to South African and Mediterranean plants were investigated by multilocus sequence analysis (MLSA) of four core housekeeping genes (*rrs*, *gyrB*, *rpoB* and *rpoD*). A total of 35 different sequence types were found for the 38 nectar bacterial isolates characterised. Phylogenetic analyses resulted in the identification of three main clades [nectar groups (NGs) 1, 2 and 3] of nectar pseudomonads, which were closely related to five intrageneric groups: *Pseudomonas oryzihabitans* (NG 1); *P. fluorescens*, *P. lutea* and *P. syringae* (NG 2); and *P. rhizosphaerae* (NG 3). Linkage disequilibrium analysis pointed to a mostly clonal population structure, even when the analysis was restricted to isolates from the same floristic region or belonging to the same NG. Nevertheless, signatures of recombination were observed for NG 3, which exclusively included isolates retrieved from the floral nectar of insect-pollinated Mediterranean plants. In contrast, the other two NGs comprised both South African and Mediterranean isolates. Analyses relating diversification to floristic region and pollinator type revealed that there has been more unique evolution of the nectar pseudomonads within the Mediterranean region than would be expected by chance. This is the first work analysing the sequence of multiple loci to reveal geno- and ecotypes of nectar bacteria.

## Introduction

Microorganisms find in plants heterogeneous and highly dynamic habitats which can differ widely with regard to nutrient availability and physico-chemical conditions at macro- and microscales [1]. Immigration history and different ecological and selective forces operating at each microhabitat can influence microbial presence and community composition [2]–[4], thus turning plant individuals into dynamic mosaics of host–microorganism assemblages.

Most previous studies on plant microbiology have focused on describing the astonishing diversity of prokaryotic and eukaryotic microorganisms occurring in the rhizosphere and the phyllosphere, and their direct and indirect effects on host fitness, community structure and dynamics, and evolutionary processes of host plants [3], [5]–[7]. The importance of other plant parts as microbial habitats remains yet underexplored, but important advances in their study have been made in recent years. In this sense, an emerging focus of plant research is the role of floral nectar as a natural habitat for microorganisms, and their ecological function. Most of these studies have dealt with nectar-dwelling yeasts [4], [8]–[15], but the role of nectar as a reservoir for bacterial diversity, and the possible influence of bacteria on plant fitness, have been also considered recently [16]–[21].

Floral nectar is complex biological fluid, mainly containing sugars and amino acids that provide a key reward to pollinators. Traditionally, nectar has been considered protected from microorganisms by a high osmotic pressure due to the elevated sugar concentration, a variety of secondary compounds, reactive oxygen species and several proteins with a defence function against microbial invasion [22]–[26]. However, some groups of yeasts and bacteria can overcome such chemical barriers and colonise floral nectar. Through their metabolic activity, these microorganisms can reduce the nutritional value of floral nectar and release different fermentation by-products, which could have a significant effect on pollinators’ foraging behaviour [9], [10], [14], [21], [27], [28]. The few yeast and bacterial nectarivorous communities studied so far seem to be characterised by low species richness and moderate phylogenetic diversity, suggesting that only some highly-specialised lineages can cope with the array of limiting factors of microbial growth found in floral nectar [12], [13], [16], [18]–[20], [22], [29]. One of the lineages of nectar-inhabiting bacteria that have overcome the chemical and osmotic barriers of nectar is the genus *Pseudomonas.*

The genus *Pseudomonas* ‘sensu stricto’ (as defined by De Vos et al. [30] comprises a diverse group of Gammaproteobacteria generally regarded as truly ubiquitous, metabolically versatile and with rather simple nutritional requirements [31]–[33]. *Pseudomonas* species thrive in most known natural environments, where they play relevant roles for the functioning of ecosystems and can establish intimate associations with animal and/or plant hosts [31], [32], [34]–[36]. Some members of this genus have been recently recovered from floral nectar of cultivated [17] and wild plants [16], [18]–[20], but a detailed analysis of the diversity and ecological significance of the nectar pseudomonads is still pending.

In this study, we explored the phylogenetic and evolutionary relationships among nectar-inhabiting pseudomonads associated to Mediterranean and South African insect-pollinated plants. To this end, a collection of 38 nectar *Pseudomonas* isolates was characterised by multilocus sequence analysis (MLSA) targeting core housekeeping genes. The relative contribution of mutation and recombination in shaping the diversity of *Pseudomonas* in floral nectar was assessed. Finally, relevant ecological information was used to infer possible evolutionary links among the identified clades. This is the first work analysing the sequence of multiple loci to reveal geno- and ecotypes of nectar bacteria.

## Materials and Methods

### Ethics Statement

Field work for this and other related studies (see below) was conducted in several publicly-owned, protected areas in Spain and South Africa, under permits issued by Dirección General de Sostenibilidad en la Red de Espacios Naturales (Consejería de Medio Ambiente, Junta de Andalucía, Spain), Dirección General de Gestión del Medio Natural (Consejería de Medio Ambiente, Junta de Andalucía, Spain), Iltmo. Ayuntamiento de Hinojos (Huelva, Spain) and Ezemvelo KZN Wildlife (KwaZulu-Natal province, South Africa). Field sampling only involved one protected plant species (*Echium gaditanum*, Boraginaceae) for which we obtained the collecting permit 1005/MDCG/mect from Dirección General de Sostenibilidad en la Red de Espacios Naturales (Consejería de Medio Ambiente, Junta de Andalucía, Spain).

### Bacterial Isolates

A collection of 38 *Pseudomonas* ‘sensu stricto’ isolates was included in the present study (Table S1). These isolates were recovered from 30 floral nectar samples of 14 phylogenetically diverse, animal-pollinated wild plant species collected in five western Mediterranean and South African plant communities (22 and 16 isolates in each region, respectively) following the methods described by Álvarez-Pérez et al. [16]. Except for *Convolvulus althaeoides* (Convolvulaceae), which seems to be a particularly well-suited host for *Pseudomonas* and other prokaryotic microorganisms [18], the number of isolates recovered from the other 13 plant species included in the present study was very similar (mean = 1.9 isolates/plant species, range = 1–3).

Thirty-five of the studied isolates were from previous studies, in which the genus *Pseudomonas* was recovered from 16.2−31.6% of nectar drops that yielded culturable bacteria [16], [18]. The remaining three isolates were recovered from new nectar samples from the same sampling sites (de Vega et al., unpublished data). All bacterial isolates were grown on trypticase soy agar (TSA; Panreac, Castellar del Vallès, Spain) plates at 25°C and stored at –20°C in Luria-Bertani (LB) broth (Difco, Sparks, MD, USA) containing 25% glycerol (Sigma-Aldrich, Madrid, Spain) until further characterisation.

### PCR Amplification and Sequencing of Selected Loci

Genomic DNA was isolated by boiling bacterial colonies in 500 µl of ultrapure deionised water at 100°C for 20 min. Cell debris was removed by centrifuging at 8000 *g* for 2 min.

Four core housekeeping genes were selected for sequence analysis: the 16S rRNA gene (*rrs*); *gyrB*, which encodes for the β subunit of the DNA gyrase; and *rpoB* and *rpoD*, which encode for the β and D subunits of the RNA polymerase, respectively. All these molecular markers are ubiquitous in bacteria and have been used in a recent comprehensive assessment of the intrageneric structure of the genus *Pseudomonas* [37].

PCR primers used for partial amplification of the studied genes are shown in Table S2. The *rrs* gene was amplified using the same reaction mixtures and PCR conditions than in Álvarez-Pérez et al. [16]. For the *gyrB*, *rpoB* and *rpoD* genes, a typical PCR contained 5 µl of NH_4_ buffer (10×, Bioline, London, UK), 1 to 2 mM MgCl_2_ (Bioline), 0.4 to 0.5 µM of each primer (Sigma-Aldrich; Table S2), 250 µM of each dNTP (Sigma-Aldrich), 1.5 to 2.5 U Biotaq DNA polymerase (Bioline), and 2 to 5 µl of DNA extract in a final volume of 50 µl. The same basic protocol was used to amplify these three genes, with some variations in the annealing temperature (Ta) and number of cycles. Following an initial denaturation step of 5 min at 94°C, 35 or 40 PCR cycles were performed (94°C for 30–60 s, Ta for 30–60 s, 72°C for 90–120 s), followed by a final extension step at 72°C for 10 min. The annealing temperature used for each primer pair can be seen in Table S2. PCR products were cleaned up with ExoSAP-IT (USB Corporation, Cleveland, OH, USA) and two-way sequenced using the ABI Prism BigDye Terminator v3.0 Ready Reaction Cycle Sequencing Kit (Applied Biosystems, Madrid, Spain) with the corresponding primers on an automated sequencer (ABI Prism 3130xl, Applied Biosystems). The nucleotide sequences determined in this work have been deposited in the GenBank database under the accession numbers KC822762 to KC822913 (see Table S1 for further details).

### Sequence Assembly and Alignment

DNA sequences were assembled and manually edited with the program Sequencher v.4.9 (Gene Codes Corporation, Inc., Ann Arbor, MI, USA) and included in multiple alignments generated by MUSCLE (http://www.ebi.ac.uk/Tools/msa/muscle/, [38]). For the *rpoD* gene, the edited DNA sequences were translated to protein sequences with the Transeq program (http://www.ebi.ac.uk/Tools/st/emboss_transeq/, [39]) before performing the MUSCLE alignment, and then back-translated to the nucleotide alignment using TranslatorX (http://translatorx.co.uk/, [40]). The resulting alignments were trimmed with BioEdit v.7.0.9.0 [41] to ensure that all sequences had the same start and end point, and analysed with Gblocks v.0.91b [42] to eliminate poorly aligned positions and divergent regions, using ‘allow gap positions = with half’, ‘minimum length of a block’ equal to 5 for the *rrs* gene and 10 for protein-encoding genes, and default settings for all other options.

### Analysis of Nucleotide Diversity

For each sequenced gene, different alleles were assigned arbitrary numbers, and unique combinations of four allele numbers (i.e. allelic profiles) were used to unambiguously define the sequence type (ST) of isolates. Molecular diversity indices and the guanine plus cytosine (G+C) content of the sequences analysed were determined using the DnaSP v.5.0 program [43]. This same software was used to perform the Tajima’s D neutrality test, which examines the neutral mutation hypothesis by using DNA polymorphism [44]. The ratio between the numbers of non-synonymous substitutions (dN; resulting in a different amino acid) and synonymous substitutions (dS; resulting in the same amino acid) was computed using the Sequence Type Analysis and Recombinational Tests (START) v.2 program [45].

### Phylogenetic Reconstruction

Phylogenetic trees for each individual gene and their concatenation (in the following order: *rrs*, *gyrB*, *rpoB* and *rpoD*) were constructed using Bayesian inference (BI) and maximum likelihood (ML) methods.

BI analyses were performed with MrBayes v.3.1.2 [46]. The simplest models of sequence evolution among those available in MrBayes that best fitted the sequence data were determined using the Akaike Information Criterion (AIC). These tests were conducted using the jModeltest v.0.1.1 package [47], and resulted in selection of a general time-reversible model with gamma-distributed rate variation across sites and a proportion of invariable sites (GTR+G+I) in all cases. Four Metropolis-coupled Markov chains were run twice for all the datasets, until average standard deviation of split frequencies fall below 0.01 (1·10^6^–2.2·10^6^ generations). The chains were sampled each 100 generations and chain temperature was set to 0.2. Fifty percent majority rule consensus trees were calculated using the *sumt* command and discarding the first 25% of the trees to yield the final Bayesian estimates of phylogeny. Posterior probabilities (PP) from the 50% majority rule consensus trees were used as estimates of robustness.

ML analyses were carried out using the online version of PhyML v.3.0 (http://www.atgc-montpellier.fr/phyml/, [48]), under the GTR+G+I model of molecular evolution, with four substitution rate categories, starting trees generated by BIONJ, and SPR tree search algorithms. Model parameters were estimated from the dataset and support for the inferred topologies was tested using 100 bootstrap replications.

The Shimodaira–Hasegawa (SH) test [49], which determines the likelihood of a dataset given alternative trees, was used to test phylogenetic congruence between trees. This analysis was performed with TREE-PUZZLE v.5.2 [50], and tree topologies with *p*-values <0.05 were considered to be incongruent with the dataset under analysis.

### Taxonomy of Nectar Isolates

In order to determine the taxonomic affiliation of nectar *Pseudomonas* isolates, the EzTaxon-e server (http://eztaxon-e.ezbiocloud.net/, last accessed 28 Mar. 2013; [51]) was used to search for neighbours among validly named bacterial species on the basis of *rrs* gene sequences.

Additionally, an alignment of concatenated (*rrs*+*gyrB*+*rpoB*+*rpoD*) sequences from nectar isolates and 34 reference strains of *Pseudomonas* (Table S3) was generated by MUSCLE and further analysed with Gblocks following the same procedure as above. A phylogenetic tree was then inferred from this alignment by the neighbour-joining (NJ) method [52], using the Molecular Evolutionary Genetics Analysis (MEGA) v.5 program [53]. Evolutionary distances were computed by the Jukes-Cantor (JC) method [54], and the rate of variation among sites was modelled by a gamma distribution, with shape parameters set at the values estimated by PhyML. All ambiguous positions were removed for each sequence pair and 1000 bootstrap replications were used to infer consensus trees. A concatenation of sequences of the studied genes corresponding to the type strain of *Cellvibrio japonicus* (Table S3) was used to root the tree. Branch support of the NJ tree was also evaluated by the ML and BI methods, which were performed as explained above. Interpretation of the resulting clades and their intrageneric relationships was in accordance with the recently published overview of genus *Pseudomonas* classification [37].

### Split Decomposition and Recombination Analyses

To assess the possibility of reticulate evolution (i.e. networked relationships pointing to incompatibilities within and between datasets), split networks of single-loci and the concatenated dataset were constructed with SplitsTree4 [55] by the Split decomposition method, using default options. For ideal data, this method results in tree-like representations, whereas network-like structures can be interpreted as possible evidence for conflicting phylogenies [56].

The possible presence of recombination in the studied genes was first assessed by the pairwise homoplasy index (PHI) test [57] as implemented in Splitstree4. Furthermore, potential recombination events were identified using the Recombination Detection Program (RDP) v.3.44 [58]. This program implements several methods for the identification of recombinant sequences and recombination breakpoints, of which we chose the seven following ones: RDP [59], BootScan [60], MaxChi [61], Chimaera [62], GeneConv [63], SiScan [64] and 3Seq [65]. Only recombination events that were identified by more than three methods were accepted as evidence of recombination and kept for detailed analyses; a similar conservative approach has been used by other authors [e.g. 66]. In all methods, sequences were considered as linear and statistical significance was set at the *p*<0.05 level, after considering Bonferroni correction for multiple comparisons. Other common settings were to require phylogenetic evidence, to polish breakpoints, to check alignment consistency, to disentangle overlapping signals and to automask sequences for optimal recombination detection. Potential major and minor parents of recombinant sequences were determined whenever possible.

Linkage disequilibrium was evaluated from allelic data using the standardised index of association (*I_A_^S^*) method, as implemented in LIAN v3.5 [67]. The null hypothesis of complete linkage equilibrium (i.e. free recombination among sequences; *I_A_^S^* = 0) was tested by both parametric and Monte Carlo methods (1000 resamplings).

The LDhat module [68] available within RDP was used to estimate recombination and mutation rates (*ρ* and *θ*, respectively) both for single loci and concatenated sequences. To estimate the initial value of *ρ*, a first preliminary run was set up with *ρ* = 30. Then, the previously determined *ρ* was used as a starting value for subsequent runs. Converging results with a reduction of confidence intervals for the estimated parameters were typically obtained with block penalty values of 30, but other values (10, 20 and 40) were also tested. All other settings were kept as default, and analyses where run with 10^6^ Monte Carlo Markov Chains (MCMC) updates including 10^5^ burn-in MCMC updates.

### Unifrac Analyses

In order to compare in a phylogenetic context the *Pseudomonas* communities found in the two floristic regions analysed (i.e. western Mediterranean and South Africa), the unweighted UniFrac test was performed using the UniFrac server (http://bmf.colorado.edu/unifrac, [69]). This test accounts for both the tree topology and the branch lengths, and tests the hypothesis that there has been more unique evolution within each environment (more branch length leading to descendants from only one environment) than would be expected by chance [69], [70]. The rooted NJ tree based on the concatenated dataset which included DNA sequences of nectar isolates and reference strains (see above) and a text file linking sequence names to the environment information −i.e. biogeographic region of origin (as shown in Table S1)− were used as inputs for the tests. UniFrac tests were performed pooling the unique branches for all the environments in the tree or considering each particular environment individually (‘all environments together’ and ‘each environment individually’ options, respectively), with 100 permutations and *p*-values corrected for multiple comparisons (Bonferroni correction).

On the other hand, we tentatively assessed possible differences in the *Pseudomonas* communities associated to different pollinators (at the order level: Coleoptera, Diptera, Lepidoptera or Hymenoptera). In this case, UniFrac analyses were performed as described above, but using the list of pollinator types under analysis (Table S4) as environment information. However, due to the limited number of *Pseudomonas* isolates recovered from most plant species, host-based UniFrac analyses were not performed.

## Results

### Sequence Variation in Core Housekeeping Genes

The genetic diversity indices calculated from the multilocus data for the whole collection of nectar isolates characterised in this study are shown in Table 1. The length of the sequences analysed ranged from 369 bp for *rpoB* to 1389 for the *rrs* gene (Table 1). From 38 isolates, 35 different haplotypes (STs) were found. The mean number of haplotypes per locus was 25.5 and ranged from 15 (for *rrs*) to 31 (*rpoD*) (Table 1). The *rpoD* gene exhibited the highest proportion of polymorphic sites (50.8%), followed by *gyrB* (36.8%) and *rpoB* (30.1%). Only 95 polymorphic sites (6.8%) were identified in the *rrs* sequences. Nucleotide diversity (π) varied widely across genes for the entire collection of nectar isolates (between 0.029 and 0.232, for *rrs* and *rpoD*, respectively; Table 1). The G+C content of the four gene fragments was similar, and ranged from 53.6% (for *rrs*) to 62.9% (for *rpoD*). Tajima’s D values did not deviate significantly from zero in any case (*p*>0.05), thus suggesting that the studied genes were not subject to selection. The ratio between the number of non-synonymous and synonymous substitutions (dN/dS) for the three protein encoding genes analysed was below 1 in all cases (Table 1).

**Table 1:** Analysis of the studied loci for the 38 nectar isolates of *Pseudomonas* characterised in this study.

Locus	Fragmentlength (bp)	Number ofhaplotypes	% Polymorphicsites*	π†	%G+C	dN/dS‡	Tajima’s D¶
*gyrB*	495	29	36.8	0.140	56.8	0.107	0.312
*rpoB*	369	27	30.1	0.101	59.8	0.056	–0.207
*rpoD*	525	31	50.8	0.232	62.9	0.511	0.434
*rrs*	1389	15	6.8	0.029	53.6	NA	1.816

* Excluding gaps and missing data.

† Nucleotide diversity (average number of nucleotide differences per site between two sequences [98]), after Jukes and Cantor correction.

‡ Ratio between the numbers of non-synonymous and synonymous substitutions (dN and dS, respectively). NA, not applicable.

¶ Tajima’s D neutrality test [44]. No significant (*p*>0.05) deviation from zero was observed for any of the studied loci.

### Phylogeny of Nectar Isolates

**Figure 1:**
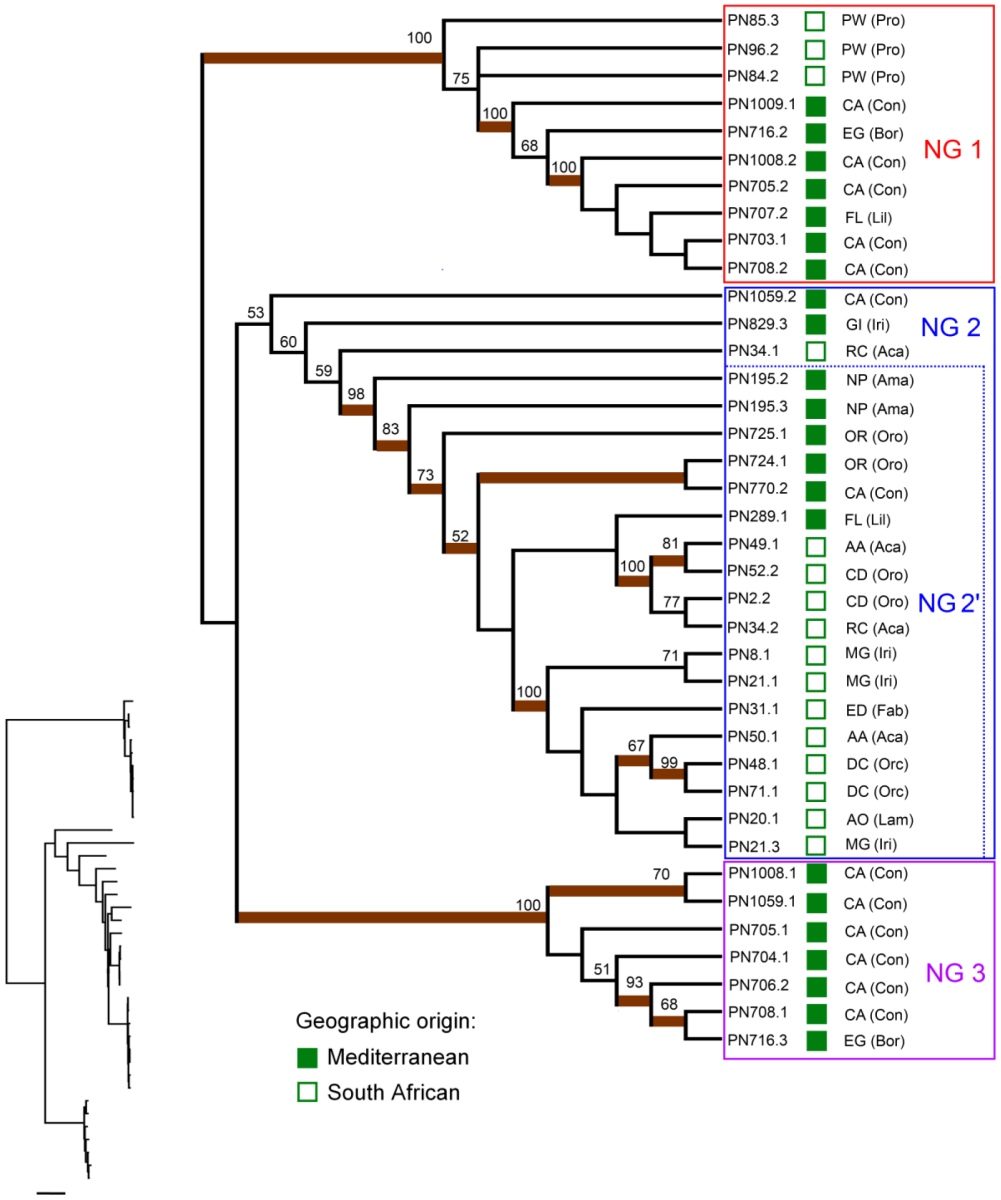
Maximum likelihood (ML) consensus tree from concatenated (*rrs* + *gyrB* + *rpoB* + *rpoD*) sequences of nectar-inhabiting *Pseudomonas* isolates. Bootstrap percentages from ML analysis are shown above lines, and clades with Bayesian posterior probabilities ≥0.9 are indicated by thick brown lines. The small phylogram is included to illustrate branch length heterogeneity (scale bar = 0.1 nucleotide substitutions per site). The geographic origin of isolates is shown on the leaves: Mediterranean, filled squares; South African, empty squares. Plant hosts and their corresponding families are also indicated, with abbreviations for the latter shown in parentheses. Insect pollinators associated to each plant species are listed in Table S4. Abbreviations for plant species names: AA: *Adhatoda andromeda*; AO: *Ajuga ophrydis*; CA, *Convolvulus althaeoides*; CD: *Cycnium adonense*; DC: *Disa crassicornis*; ED: *Eriosema distinctum*; EG: *Echium gaditanum*; FL: *Fritillaria lusitanica*; GI: *Gladiolus illyricus*; MG: *Moraea graminicola*; NP: *Narcissus papyraceus*; OR, *Orobanche ramosa*; PW, *Protea welwitschii*; RC, *Ruellia cordata*. Abbreviations for plant families: Aca, Acanthaceae; Ama, Amaryllidaceae; Bor, Boraginaceae; Con, Convolvulaceae; Fab, Fabaceae; Iri, Iridaceae; Lam, Lamiaceae; Lil: Liliaceae; Orc, Orchidaceae; Oro, Orobanchaceae; Pro, Proteaceae.

The trees based on the concatenation of the four genes analysed (2778 bp in total) grouped the 38 nectar isolates into three major clades, hereafter referred to for convenience as ‘nectar groups’ (NGs) 1, 2 and 3. NGs 1 and 3 were well supported by both ML and BI analyses (100% ML bootstrap support and >0.9 BI posterior probability, in both cases; Fig. 1). ML and BI support for NG 2 was weak, although it included a highly supported subclade (NG 2′, Fig. 1). NGs 1 and 2 comprised nectar isolates recovered from both South African and Mediterranean plants, while NG 3 included exclusively isolates from the Mediterranean (6 of the 13 isolates recovered from *C. althaeoides* and 1 of the 2 isolates from *E.* *gaditanum*).

Individual ML trees constructed from each independent gene also supported NGs 1 and 3, but not NG 2 nor 2′ (except for *rpoD*; Fig. S1). Other smaller subgroups could be recognised within NG 2 but, to simplify subsequent analyses, these were not considered.

The NGs defined from phylogenetic analyses displayed extensive variation in their nucleotide diversity indices for the studied genes, with the highest values being observed for NG 2 and the lowest for NG 3 (Table S5).

The results of the SH test performed on individual gene sequences and on the concatenated dataset showed that most ML trees were incongruent with each other but, except for *rpoB*, were not significantly different from the ML tree based on the alignment of concatenated sequences (Table 2). Furthermore, all datasets supported the tree obtained by the BI method for the concatenation of individual loci (Table 2). Therefore, although the studied genes may not exhibit the same evolutionary history, in general terms the trees based on the alignment of concatenated sequences did not contradict the information brought by each individual locus.

**Table 2:** *P*-values determined using the Shimodaira-Hasegawa (SH) test of tree topologies.

Tree†	Dataset‡				
	*gyrB*	*rpoB*	*rpoD*	*rrs*	Concatenated
ML method					
*gyrB*	1.0000	0.0010*	<0.0001*	<0.0001*	0.0380*
*rpoB*	<0.0001*	1.0000	<0.0001*	<0.0001*	<0.0001*
*rpoD*	<0.0001*	0.0100*	1.0000	0.0030*	0.0480*
*rrs*	<0.0001*	<0.0001*	<0.0001*	1.0000	<0.0001*
Concatenated	0.1900	0.0150*	0.0890	0.0660	1.0000
BI method					
Concatenated	0.2810	0.2580	0.2300	0.1190	1.0000

† Comparisons are based on the trees inferred by the maximum likelihood (ML) and Bayesian inference (BI) methods for individual loci or a concatenation of them.

‡ Tree topologies not supported (*p*<0.05) by the corresponding dataset are marked by an asterisk.

### Network and Recombination Analyses

The information obtained for all individual genes and the concatenated dataset by split decomposition suggested some degree of reticulation (i.e. conflicting phylogenetic signals) for *rrs*, but not for *gyrB*, *rpoB* and *rpoD* datasets or the concatenation of individual sequences (Fig. S2).

The PHI test showed evidence of recombination for *rrs* and the concatenation of genes (*p*<0.001, in both cases), while significant intragenic recombination (*p*<0.05, detection by >3 methods) was only detected in RDP analyses for *rrs* and *gyrB*, with a single recombination event identified for each gene (Table 3). Nearly all recombinant sequences for both genes corresponded to NG 3 isolates (7 of 9 for *rrs* and 6 of 7 for *gyrB*; Table 3), with only a few recombinants belonging to NG 2 isolates (2 of 9 and 1 of 7, respectively). Regarding region of origin, all recombinants except one originated from the Mediterranean area. The putative major parents of all recombinant sequences were in NG 2′, while the minor parent belonged to NG 1 for *rrs* and remained unknown for *gyrB*.

**Table 3:** Significant recombination events detected using the Recombination Detection Program (RDP) v.3.4.2.

Recombination event	Gene	Recombinants (NG*)	Methods	Parents (NG)	
				Major	Minor
1	*rrs*	PN34.1 (NG 2), PN704.1 (NG 3), PN705.1 (NG 3), PN706.2 (NG 3), PN708.1 (NG 3), PN716.3 (NG 3), PN829.3 (NG 2), PN1008.1 (NG 3), PN1059.1 (NG 3)	RDP, GENECONV, BootScan,MaxChi, Chimaera, SiScan, 3Seq	PN195.2 (NG 2′)	PN708.2 (NG 1)
2	*gyrB*	PN704.1 (NG 3), PN705.1 (NG 3), PN706.2 (NG 3), PN708.1 (NG 3), PN716.3 (NG 3), PN1008.1 (NG 3), PN1059.2 (NG 2)	MaxChi, Chimaera, SiScan, 3Seq	PN289.1 (NG 2′)	Unknown

* NG: nectar group, as determined in phylogenetic analyses.

The four loci analysed displayed per site recombination/mutation rate ratios (*ρ*/*θ_w_*) below 1 (between 0.232 and 0.624, for *rpoD* and *gyrB* respectively; Table S6), suggesting that mutation occurred more often than recombination in these genes. Accordingly, a *ρ*/*θ_w_* ratio <1 was also observed for the concatenated dataset (0.190, Table S6).

When the null hypothesis of linkage equilibrium was tested for the 38 nectar isolates and for different groups (defined by geographic or phylogenetic criteria; Table 4), it was rejected in all cases except for NG 3 isolates. Most of these groups also displayed per site *ρ*/*θ_w_* ratios <1 for the concatenated dataset but, on the contrary, *ρ*/*θ_w_* ratios >1 were observed for NGs 2 and 3 (Table 4).

**Table 4:** Linkage equilibrium analysis and recombination and mutation indices for the nectar isolates.

Group of isolates	Linkage equilibrium analysis				Recombination and mutation indices‡		
			*p*-value†				
	*n* *	*I_A_^S^*	Parametric	Monte Carlo	*ρ* ¶ (×10^−3^)	*θ_w_* § (×10^−2^)	*ρ/θ_w_* ▴
All isolates	38	0.2977	<0.001	<0.001	8.028 (6.81–9.32)	4.221	0.190
Biogeographic groups							
Mediterranean	22	0.3655	<0.001	<0.001	5.904 (4.871–7.191)	4.791	0.123
South African	16	0.3260	<0.001	<0.001	0.010 (0.010–0.020)	4.800	<0.001
Phylogenetic clusters							
NG 1	10	0.2085	<0.001	0.012	0.070 (0.010–0.250)	1.175	0.006
NG 2	21	0.3165	<0.001	<0.001	49.810 (39.16–62.45)	3.899	1.278
NG 2′	18	0.3179	<0.001	<0.001	17.350 (13.49–24.66)	2.882	0.602
NG 3	7	0.0867	NS	NS	26.040 (18.99–35.15)	0.896	2.907

* Number of isolates included in the corresponding group.

† The null hypothesis of complete linkage equilibrium (*I_A_^S^* = 0) was tested by both parametric and Monte Carlo methods (1000 resamplings). NS: not significant *p*-value.

‡ Based on the concatenated dataset (*rrs*+*gyrB*+*rpoB*+*rpoD*).

¶ Rho per site (lower−upper bound, 95^th^ percentiles).

§ Theta per site (Watterson estimator).

▴ Rho/theta per site.

### Taxonomy of the Nectar Pseudomonads

Searches on the EzTaxon-e server database of almost complete *rrs* gene fragments confirmed the assignment of all the studied nectar isolates to the genus *Pseudomonas* ‘sensu stricto’. However, a conclusive identification to the species level was not possible as all query sequences returned several hits with similarity values above 97% (Table S7), which is a conservative threshold commonly taken for species delimitation in bacteria [71], [72].

**Figure 2:**
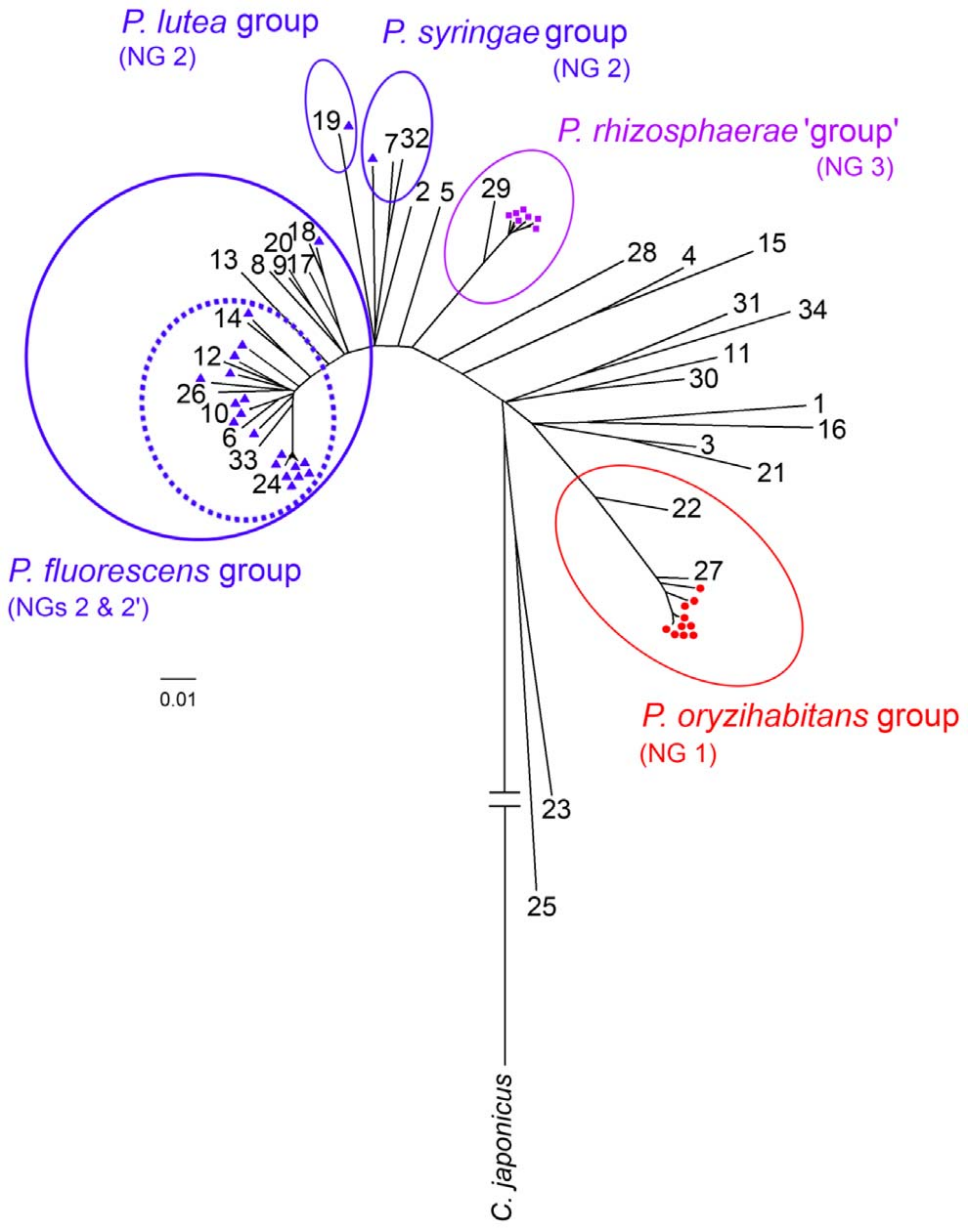
Unrooted neighbour-joining (NJ) consensus tree, based on phylogenetic analysis of concatenated (*rrs* + *gyrB* + *rpoB* + *rpoD*) sequences, displaying the relationships of nectar-inhabiting isolates (shown as red filled circles [nectar group (NG) 1], blue filled triangles [NG 2] or violet filled squares [NG 3]) and reference strains of *Pseudomonas* ‘sensu stricto’ species (shown as numbers, see below). Nectar isolates belonging to subgroup NG 2′ are shown within a dotted circle. Evolutionary distances were computed using the Jukes-Cantor method and are in the units of number of base substitutions per site. *Cellvibrio japonicum* Ueda107^T^ was used as an outgroup. A complete version of this figure is provided as supporting information (Figure S3). Numeric codes for reference strains shown in the tree: 1, *P. aeruginosa* DSM 50071^T^; 2, *P. agarici* LMG 2112^T^; 3, *P.alcaliphila* AL15-21^T^; 4, *P. anguilliseptica* NCIMB 1949^T^; 5, *P. asplenii* ATCC 23835^T^; 6, *P. azotoformans* IAM 1603^T^; 7, *P. cannabina* CFBP 2341^T^; 8, *P. chlororaphis* DSM 50083^T^; 9, *P. corrugata* ATCC 29736^T^; 10, *P. extremorientalis* KMM 3447^T^; 11, *P. flavescens* B62^T^; 12, *P. fluorescens* IAM 12022^T^; 13, *P. fragi* ATCC 4973^T^; 14, *P. gessardii* CIP 105469^T^; 15, *P. guineae* LMG 24016^T^; 16, *P. indica* IMT37^T^; 17, *P. jessenii* CIP 105274^T^; 18, *P. koreensis* KACC 10848^T^; 19, *P. lutea* OK2^T^; 20, *P. mediterranea* CFBP 5447^T^; 21, *P. oleovorans* IAM 1508^T^; 22, *P. oryzihabitans* IAM 1568^T^; 23, *P. pachastrellae* KMM 330^T^; 24, *P. palleroniana* CFBP 4389^T^; 25, *P. pertucinogena* IFO 14163^T^; 26, *P. poae* DSM 14936^T^; 27, *P. psychrotolerans* C36^T^; 28, *P. putida* IAM 1236^T^; 29, *P. rhizosphaerae* IH5^T^; 30, *P. straminea* IAM 1598^T^; 31, *P. stutzeri* ATCC 17588^T^; 32, *P. syringae* NCPPB 281^T^; 33, *P. tolaasii* LMG 2342^T^; 34, *P. xanthomarina* KMM 1447^T^.

NJ analysis of the concatenated *rrs*, *gyrB*, *rpoB* and *rpoD* sequences of the strains characterised in this study and type strains of *Pseudomonas* allowed the classification of the nectar isolates in different lineages, groups and subgroup (Figs. 2 and S3, and Table S8). All NG 1 isolates clustered with *P. psychrotolerans*, which belongs to the *P. oryzihabitans* group. On the other hand, isolates from NGs 2 and 3 clustered within the *P. fluorescens* lineage, but were close to different taxonomic groups: whereas NG 2 isolates were related to type strains from the *P. fluorescens*, *P. lutea* and *P. syringae* groups, all NG 3 isolates clustered with *P. rhizosphaerae*. NG 2′ was composed by a subset of isolates from NG 2 which clustered with reference sequences from the *P. fluorescens* and *P. gessardi* subgroups (Table S8). In general, these groupings were supported by high bootstrap values (both in NJ and ML analyses) and BI posterior probabilities (Fig. S3).

### Association between the Habitat and Phylogeny of Nectar Isolates

The results of the overall UniFrac significance test (‘all environments together’ option) indicated that there was significant (*p*≤0.01) clustering of nectar isolates in the phylogenetic tree on the basis of their biogeographic origin (Mediterranean *vs.* South African), and that these clusters represented a significant amount of unique branch length. In contrast, no relationship was found between the phylogeny of the nectar isolates and the array of pollinators associated to their host plants.

A subsequent analysis of Unifrac distances determined for individual habitat types (‘each environment individually’ option) confirmed the significant clustering of the nectar isolates associated to Mediterranean plants (*p*≤0.01). On the contrary, South African isolates were not associated by more unique branch length than expected.

## Discussion

Microbial diversity arises and is maintained through an intricate interplay of ecological and genetic factors [31], [73]. In the case of *Pseudomonas*, it seems that a long evolutionary history and continual exposure to a range of spatially and temporally complex environments have resulted in an astonishing diversity of genetic lineages and ecotypes [31], some of which are well adapted to thrive in close association to plants [35], [74]–[76]. In particular, some pseudomonads can tolerate relatively high sugar concentrations and thus colonise the sugary exudates of plants, including floral nectar [16], [19], [20] (but see Ref. [77]).

Following an MLSA approach, in this study we aimed to disclose the genetic diversity and phylogenetic affiliation of nectar *Pseudomonas* isolates recovered from different floristic regions. Subsequent analyses allowed us to assess the relative importance of mutation and recombination as evolutionary forces underlying the observed diversity of genotypes, and to propose putative ecotypes of nectar pseudomonads. All these aspects are discussed in the following paragraphs.

### Genetic Diversity in a Context of Extensive Clonality

A first result of the present investigation was the high genetic diversity found for all studied loci, which resulted in the identification of 35 MLSA sequence types among the 38 isolates analysed. Although sequencing of additional loci could have resulted in a greater discrimination of isolates, it has been recently demonstrated that the set of four core housekeeping genes included in our MLSA scheme provides enough resolution at the species level [37]. Furthermore, except for a few *Pseudomonas* species (such as *P. aeruginosa*; http://pubmlst.org/paeruginosa/), there is not yet any consensus on which markers should be used for typing isolates of this genus.

Despite the high diversity of STs observed, linkage disequilibrium analyses yielded *I_A_^S^* values significantly greater than zero for the whole collection of nectar isolates and most groups defined by biogeographic or phylogenetic criteria, all of which is suggestive of predominantly clonal population structure. The exception was NG 3, for which the null hypothesis of linkage equilibrium could not be rejected. Interestingly, this latter group only included *Pseudomonas* isolates recovered from the floral nectar of Mediterranean plants, and mainly associated to the insect-pollinated plant *C. althaeoides*. *Convolvulus althaeoides* is a nitrophilous ruderal plant, most commonly found on roadsides and disturbed patches in the Mediterranean and Macaronesic regions (but also occasionally present as an introduced species in North America), and which is visited by a wide array of insects (see Table S4). Plants of *C. althaeoides* located a few meters apart harboured communities of three different *Pseudomonas* groups, and a high diversity of other microorganisms (as shown by Álvarez-Pérez and Herrera [18]), indicating that this plant species is an interesting taxa to study nectar microbial populations.

High genetic diversity despite prevailing or exclusive clonality has been detected for different *Pseudomonas* species, such as *P. stutzeri* [78] and *P. syringae* [79], and for fluorescent pseudomonads that protect plants from soil-borne pathogens through the production of antifungal compounds [80], [81]. A similar pattern of high genetic diversity in mostly clonal microorganisms has also been recently revealed for the nectar yeast *Metschnikowia gruessii*, an observation which was attributed to microsite-dependent, divergent selection [82]. Environmental heterogeneity coupled with variable, patch-specific selective pressures would favour the long-term persistence of different clonal lineages of nectar microorganisms across different microsites, thus eventually maintaining overall genotypic diversity [82].

### The Nectar Pseudomonads are Phylogenetically Diverse

The combined analysis of multiple house-keeping genes strongly suggested extensive phylogenetic diversity and several origins for the *Pseudomonas* lineages adapted to nectar conditions. Most nectar isolates (NGs 2 and 3) clustered in the consensus trees derived from multilocus sequence data with several different species belonging to the *P. fluorescens* lineage, which represents the largest intragenic division in terms of species number and includes different groups and subgroups for which the relative positions are not fully resolved [37], [83]. On the other hand, NG 1 isolates clustered with *P. psychrotolerans*, which belongs to the *P. oryzihabitans* group. Together with *P. luteola*, *P. pachastrellae* and *P. pertucinogena*, this latter group has been characterised as the most phylogenetically distant from all other members of the genus *Pseudomonas* [37].

Although the nectar isolates included in this study displayed high similarity in their *rrs* gene sequence to previously described *Pseudomonas* species (Table S7), some of them clustered apart from reference strains in the phylogenetic trees (Figs. 2 and S3). Nevertheless, DNA-DNA hybridisations and other complementary tests commonly used in polyphasic taxonomy would be required to confirm the phylogenetic independence of those nectar lineages and, eventually, to propose the recognition of novel bacterial taxa.

### Signatures of Recombination among the Nectar Pseudomonads

Diversifying selection needs not to be the only or even the main mechanism allowing clonal bacteria to retain genetic variability, and homologous recombination can also contribute significantly to the maintenance of genomic variation in the face of clonality [84]. Nevertheless, the role of homologous gene exchange in many microbial species remains unclear, as most published studies have focused on pathogenic microorganisms [85].

In the present study, the observation of incongruences in the phylogenies inferred from partial sequences of the individual genes included in the MLSA scheme suggested the possible occurrence of recombination events among the nectar pseudomonads. This suggestion was further confirmed in some cases (such as the *rrs* and *gyrB* genes) by split decomposition, the PHI test and/or RDP analyses. However, the number of recombination events detected in RDP analyses of individual loci was low, and mostly affected NG 3 isolates. On the other hand, the *ρ*/*θ_w_* values indicated that mutation occurred more often than recombination in all the genes under study. A similar result (*ρ*/*θ_w_* <1) was observed in the analysis of the concatenated dataset for the whole collection of nectar isolates and most biogeographic and phylogenetic groups, but not for NGs 2 and 3. Nevertheless, in the case of NG 2 the effect of recombination was only limited to a few isolates (Table 3), and seemed to be insufficient to erase all clonal structure, as evidenced by linkage disequilibrium analysis. Therefore, whereas NGs 1 and 2 seem to be mostly clonal complexes, the relative contribution of recombination is predominant over mutation in the evolution of NG 3.

Contrasting recombination patterns among closely related species or phylotypes have also been inferred for other bacteria [86]–[89] and eukaryotic microorganisms [90], [91]. Unfortunately, comparison with bacterial taxa of similar ecology than our set of isolates could not be performed, as this is the first time that MLSA is applied to the study of nectar prokaryotes. The following two aspects, however, should be kept in mind when interpreting the results of the present study. Firstly, although our recombination analyses were based on a modest number of isolates, which presumably resulted in reduced statistical power, substantial evidence of recombination was found. And secondly, subpopulations of nectar pseudomonads subjected to differential local adaptation and/or genetic drift might have been lumped together, which can lead to an underestimation of the actual recombination rate [85]. In this regard, the identification of ecologically differentiated populations of microorganisms inhabiting distinct niches and potentially differing in their patterns of homologous recombination (e.g. because of adaptive evolution or environmental constraints) could help to avoid confounding effects which affect the estimation of recombination rates [85].

### Identifying Novel *Pseudomonas* Ecotypes in Floral Nectar

Ecotypes, defined as cohesive groups of organisms that show a history of coexistence as separate, ecologically distinct lineages (as inferred from community phylogeny or other sequence-based approach) and a prognosis for future coexistence (as inferred from their ecological distinctness), are the fundamental units of bacterial ecology and evolution [92]. However, most classification systems in microbiology focus on physiological and/or genotypic traits, overlooking potential differences in ecological roles between microorganisms.

Despite their close relationship to already described species, some of the clades of nectar microorganisms identified in this study by MLSA seem to represent phylogenetically independent clusters within the genus *Pseudomonas* which share the ability to inhabit floral nectar. Furthermore, the results of the UniFrac significance tests revealed there has been more unique evolution of the nectar pseudomonads within the Mediterranean region than would be expected by chance.

The question which arises is if the aforementioned results are relevant enough to grant the phylogenetically-defined groups of nectar pseudomonads the status of ecotypes.

Both for NGs 1 and 2, the answer to this latter question is unclear, as within each of these groups the isolates clustered together regardless of their biogeographic origin (i.e., nectar samples collected more than 8000 km apart harboured the same *Pseudomonas* lineages) and type of pollinator. On the contrary, despite the relative low number of isolates analysed, our results suggest that NG 3 may represent a putative novel ecotype, as it is a phylogenetically well defined cluster of *Pseudomonas* isolates inhabiting the nectar of insect-pollinated Mediterranean plants and sharing a substantial level of recombination. In our view, this interesting possibility should be readdressed by future investigations on the microbial ecology of Mediterranean insect-pollinated flowers.

### Final Remarks: Floral Nectar as a Reservoir of Microbial Diversity

Our understanding of microbial biodiversity associated to flowers in natural habitats is still in an early stage, but in the last years an increasing interest has emerged with respect to the importance of nectar as a reservoir of novel microbial species. Several novel bacteria and yeast species inhabiting floral nectar have been described so far (e.g. [93]–[96]) and the number of new described species is expected to increase as a higher number of nectars from different angiosperm lineages are sampled.

Part of the recent upsurge in nectar microbiology research stems from the realisation that nectar microbes can play important ecological roles through their effects on plant- pollinator [9]–[11], [21] and plant-pathogen [29], [97] interactions, two key elements in the functioning of terrestrial ecosystems. In view of recent demonstrations of the ecological roles of nectar microbes and their widespread occurrence, it is likely that nectar microbial ecology may become a hot topic in the next years. Novel approaches will help to reveal the mechanisms that underlie biodiversity patterns in plant-associated microbial organisms and elucidate the factors controlling the presence of particular nectar microbe lineages in any particular locality. These advances will help to understand part of the complex hidden world of microbes, really far from that which Linnaeus described for the first time under the single taxon *Chaos infusorium*.

## Supporting Information

Figure S1 Maximum likelihood (ML) trees inferred from *rrs*, *gyrB*, *rpoB* and *rpoD* sequences of 38 nectar-inhabiting *Pseudomonas* isolates. The scale bar represents the number of nucleotide substitutions per site. Bootstrap values greater than 50% are shown next to lines, and nodes supported by Bayesian posterior probabilities ≥0.9 are indicated by asterisks. The positions of the nectar groups (NGs) obtained by phylogenetic analysis of the concatenated dataset (as defined in Fig. 1, see main text) and supported by the corresponding single gene tree are indicated by boxes.
(PDF)

Figure S2 Split decomposition analysis of *rrs*, *gyrB*, *rpoB* and *rpoD* sequences, and a concatenation of the four loci for the nectar-inhabiting *Pseudomonas* isolates characterised in this study.
(PDF)

Figure S3 Neighbour-joining (NJ) consensus tree, based on concatenated (*rrs* + *gyrB* + *rpoB* + *rpoD*) sequences, showing the relationships of nectar-inhabiting isolates and reference (type) strains of *Pseudomonas* ‘sensu stricto’ species. Evolutionary distances were computed using the Jukes-Cantor method and are in the units of number of nucleotide substitutions per site. There were a total of 2601 positions in the final dataset; all positions containing gaps and missing data were eliminated. Node support values (NJ bootstrap percentages, 1000 replicates) ≥50% are shown next to the branches. Clades supported by the Maximum Likelihood (ML, ≥90% bootstrap) and Bayesian Inference (BI, ≥0.9 posterior probability) methods are indicated by filled squares and asterisks, respectively. MrBayes and PhyML settings were as explained in the main text, but using a symmetrical model of sequence evolution with gamma-distributed rate variation across sites and a proportion of invariable sites (SYM+G+I) for phylogenetic inference by the BI method. *Cellvibrio japonicum* Ueda107^T^ was used as an outgroup to root the tree. GenBank accession numbers for nectar isolates are shown in Table S1, and those corresponding to reference strains in Table S3.
(PDF)

Table S1 Details of the *Pseudomonas* ‘sensu stricto’ isolates characterised in this study.
(PDF)

Table S2 PCR primers used in this study.
(PDF)

Table S3 List of reference strains included in phylogenetic analyses.
(PDF)

Table S4 List of pollinator types for the plant species sampled.
(PDF)

Table S5 Analysis of the studied loci for the nectar groups (NGs) of pseudomonads identified in phylogenetic analyses.
(PDF)

Table S6 Recombination and mutation indices of the studied loci.
(PDF)

Table S7 Nearest neighbours on the basis of 16S rRNA (*rrs*) gene sequences among validly named bacterial species of all nectar strains characterised in this study, as obtained through the EzTaxon-e server.
(PDF)

Table S8 Tentative classification of the nectar-inhabiting *Pseudomonas* characterised in this study.
(PDF)
