# Supplementary figures and images for: Multilocus Sequence Analysis of Nectar Pseudomonads Reveals High Genetic Diversity and Contrasting Recombination Patterns
Source: PLoS One. 2013 Oct 8;8(10):e75797. doi: 10.1371/journal.pone.0075797 (PMC3792982; doi:10.1371/journal.pone.0075797)

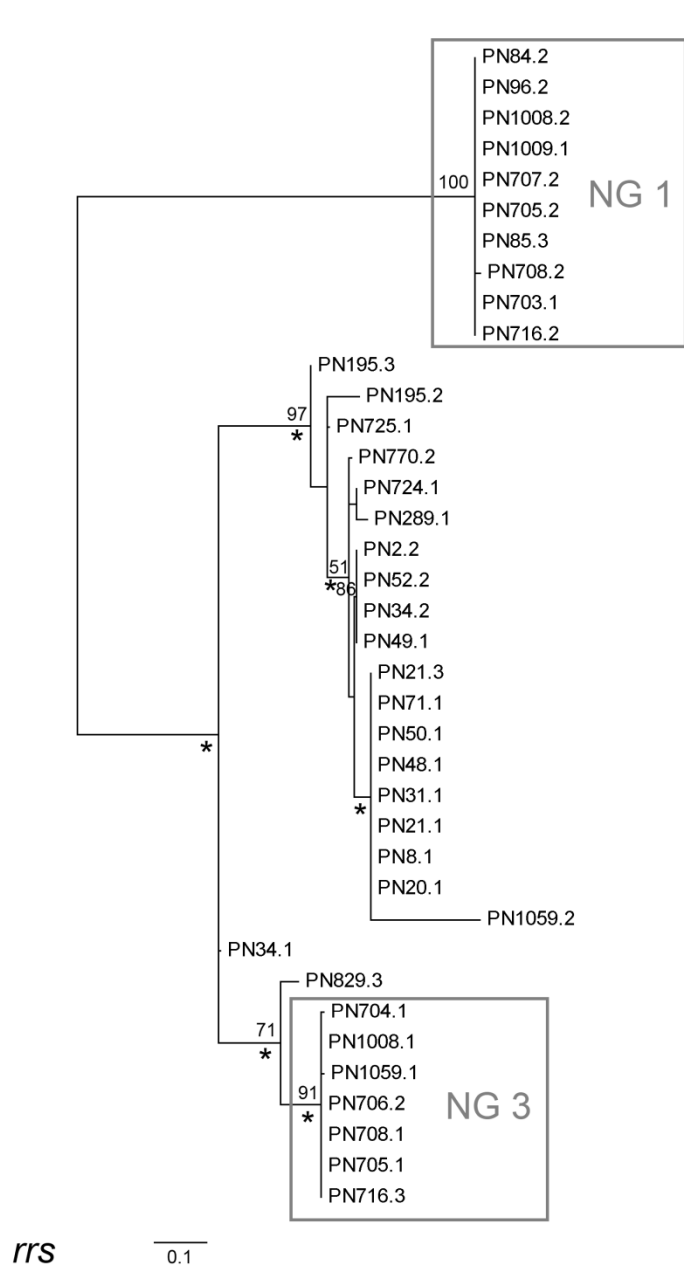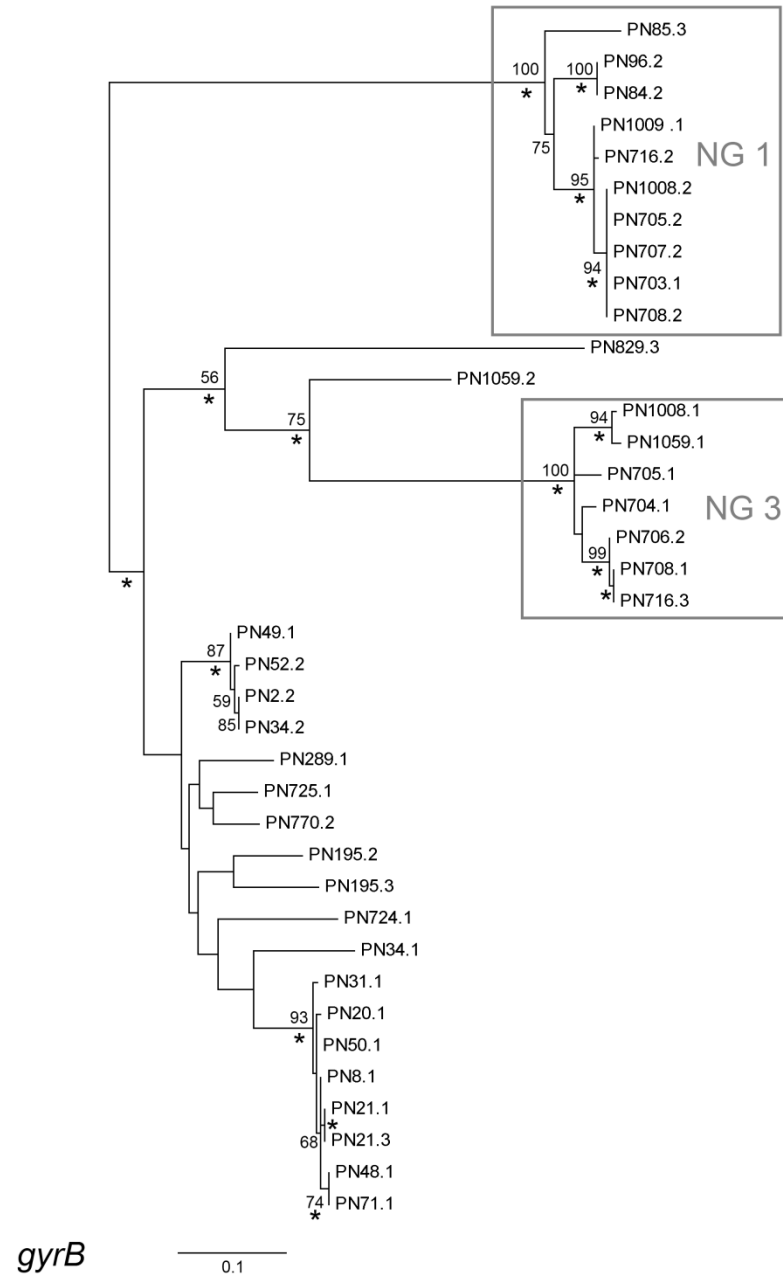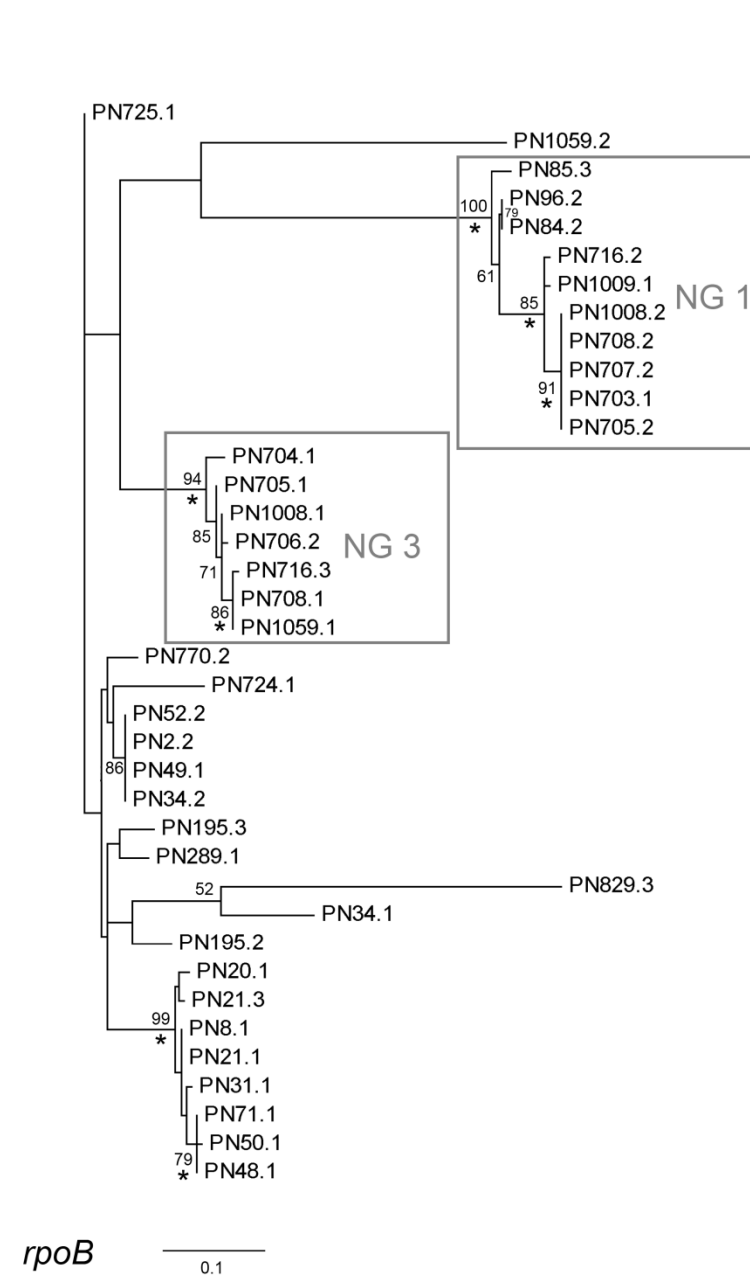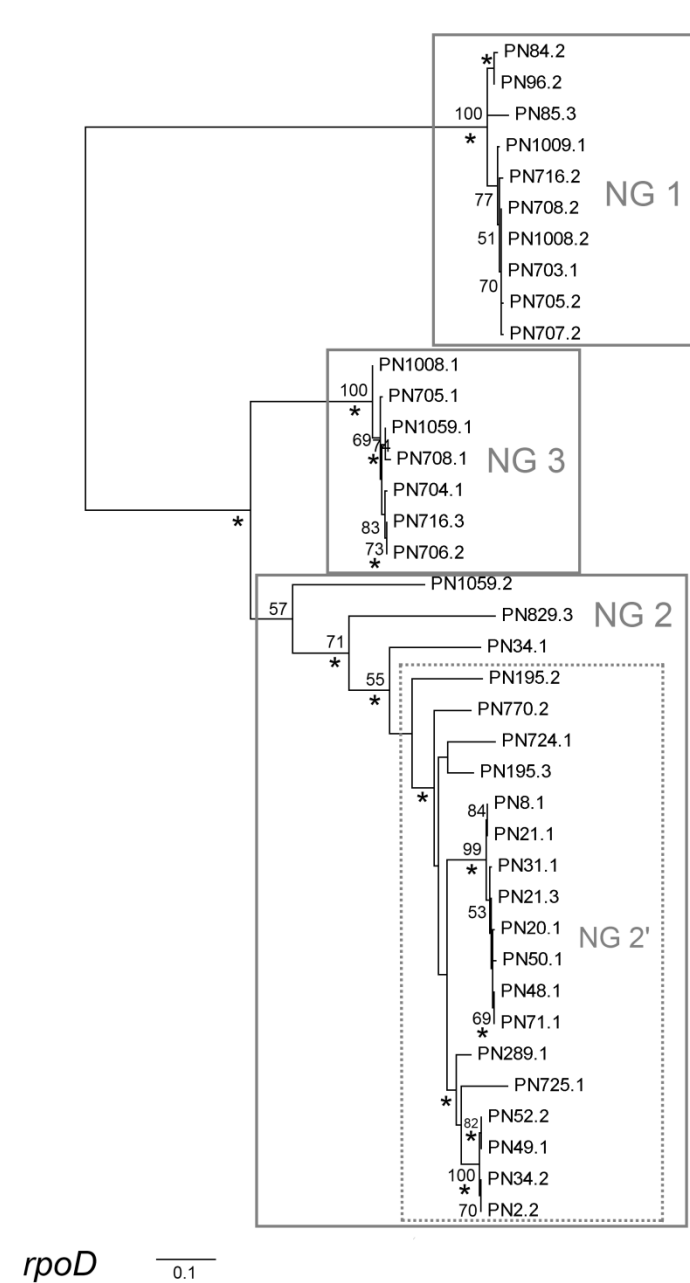

Supplement: Figure S1 — Maximum likelihood (ML) trees inferred from rrs, gyrB, rpoB and rpoD sequences of 38 nectar-inhabiting Pseudomonas isolates. The scale bar represents the number of nucleotide substitutions per site. Bootstrap values greater than 50% are shown next to lines, and nodes supported by Bayesian posterior probabilities ≥0.9 are indicated by asterisks. The positions of the nectar groups (NGs) obtained by phylogenetic analysis of the concatenated dataset (as defined in Fig. 1, see main text) and supported by the corresponding single gene tree are indicated by boxes. (PDF) [file pone.0075797.s001.pdf]

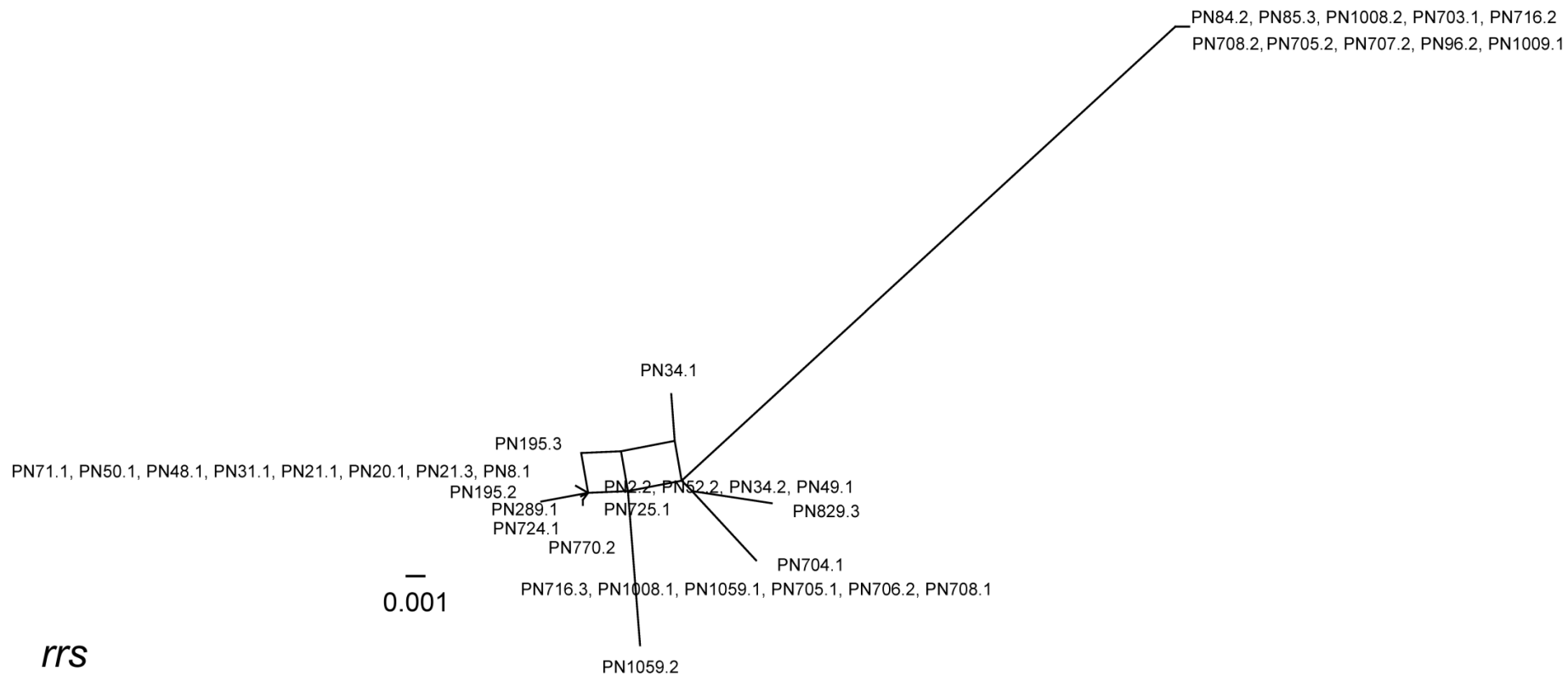

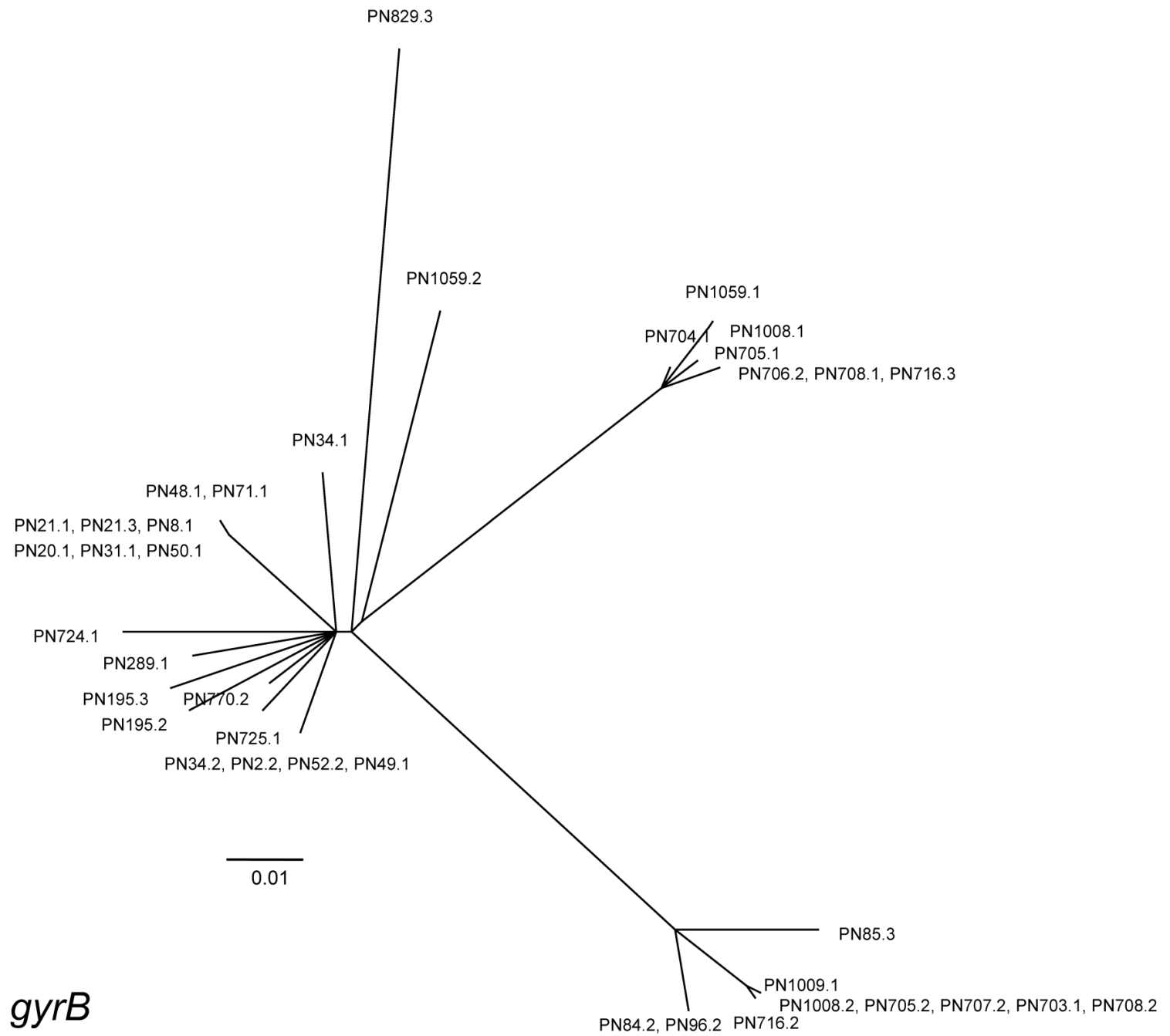

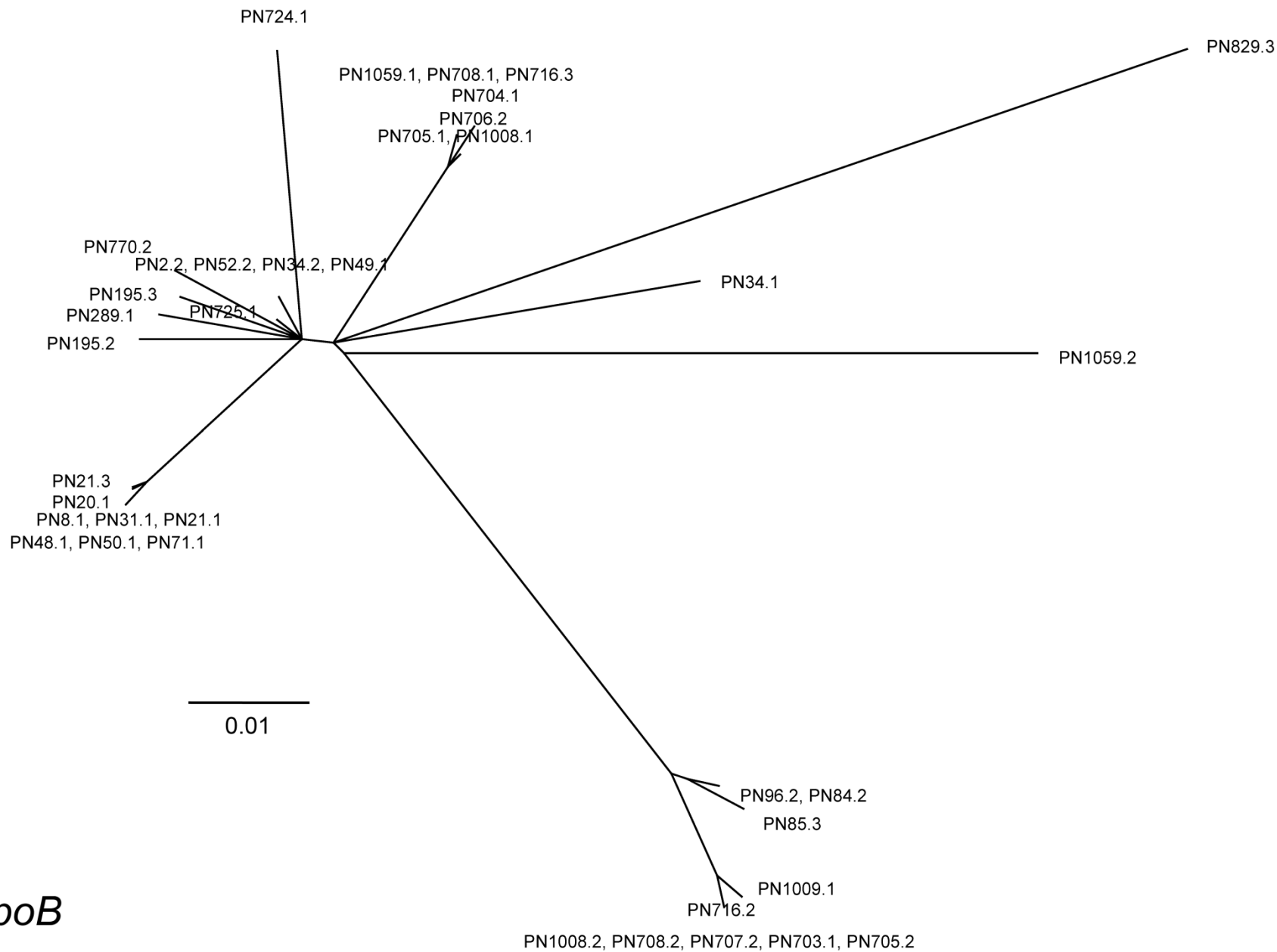

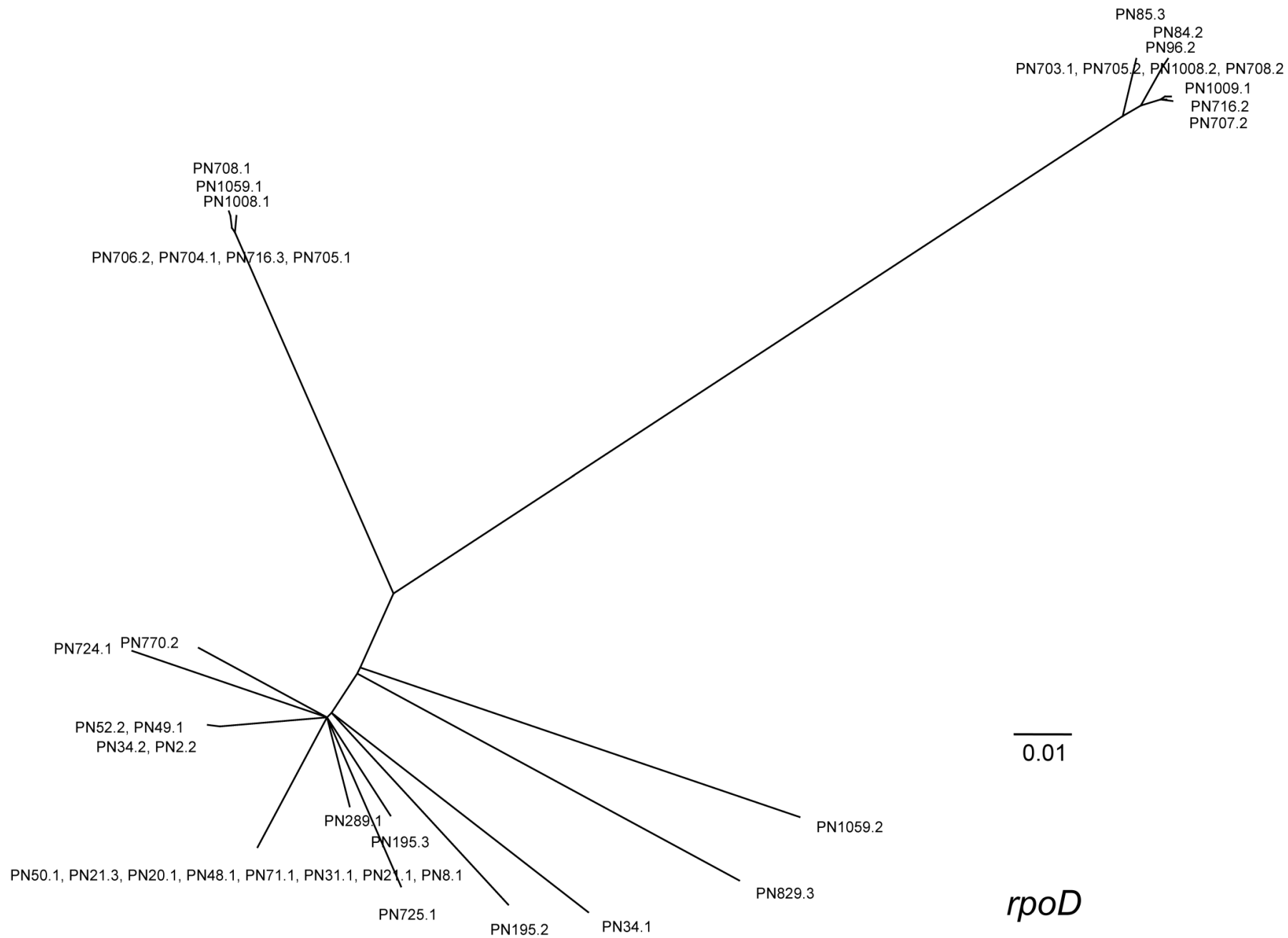

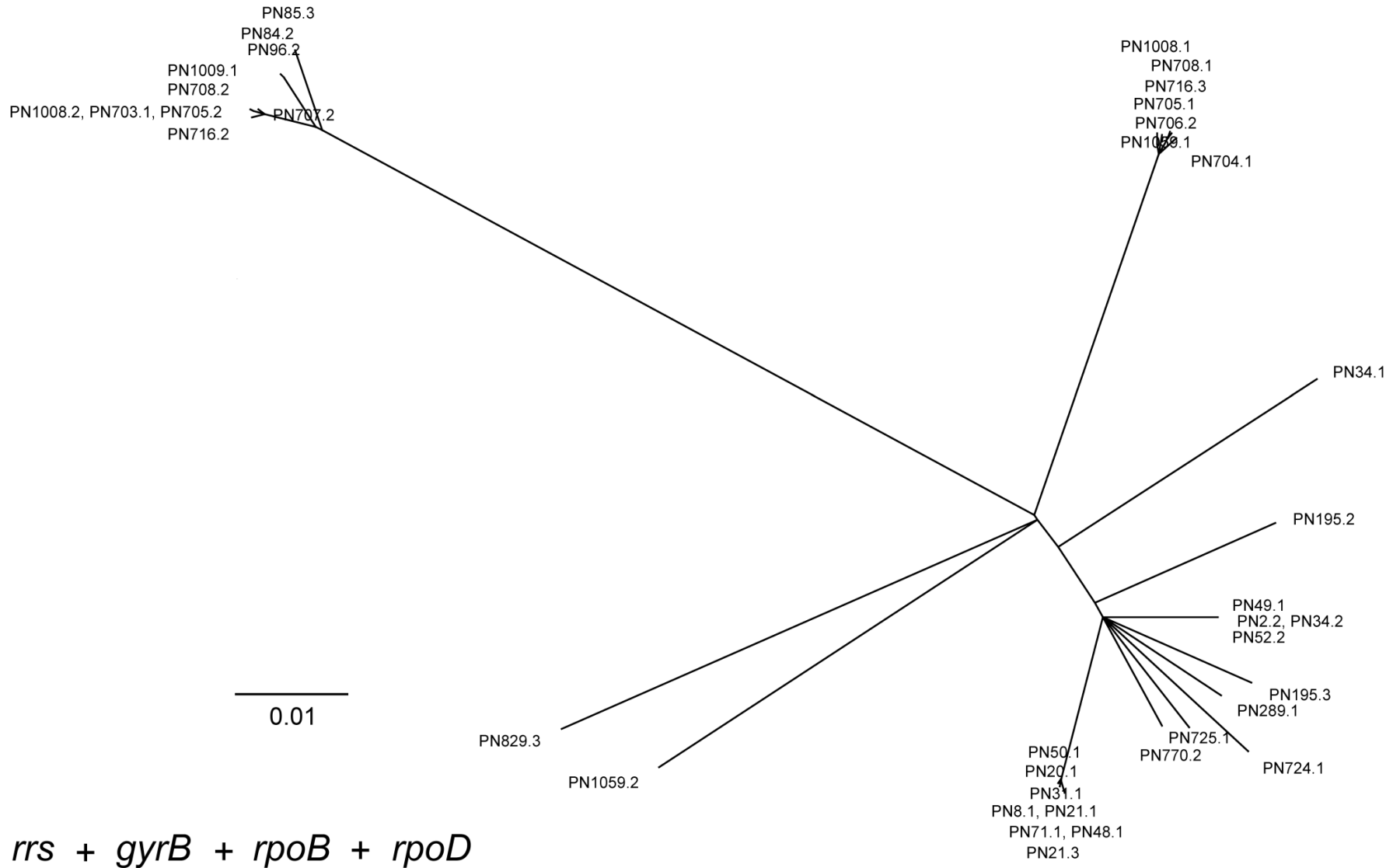

Supplement: Figure S2 — Split decomposition analysis of rrs, gyrB, rpoB and rpoD sequences, and a concatenation of the four loci for the nectar-inhabiting Pseudomonas isolates characterised in this study. (PDF) [file pone.0075797.s002.pdf]

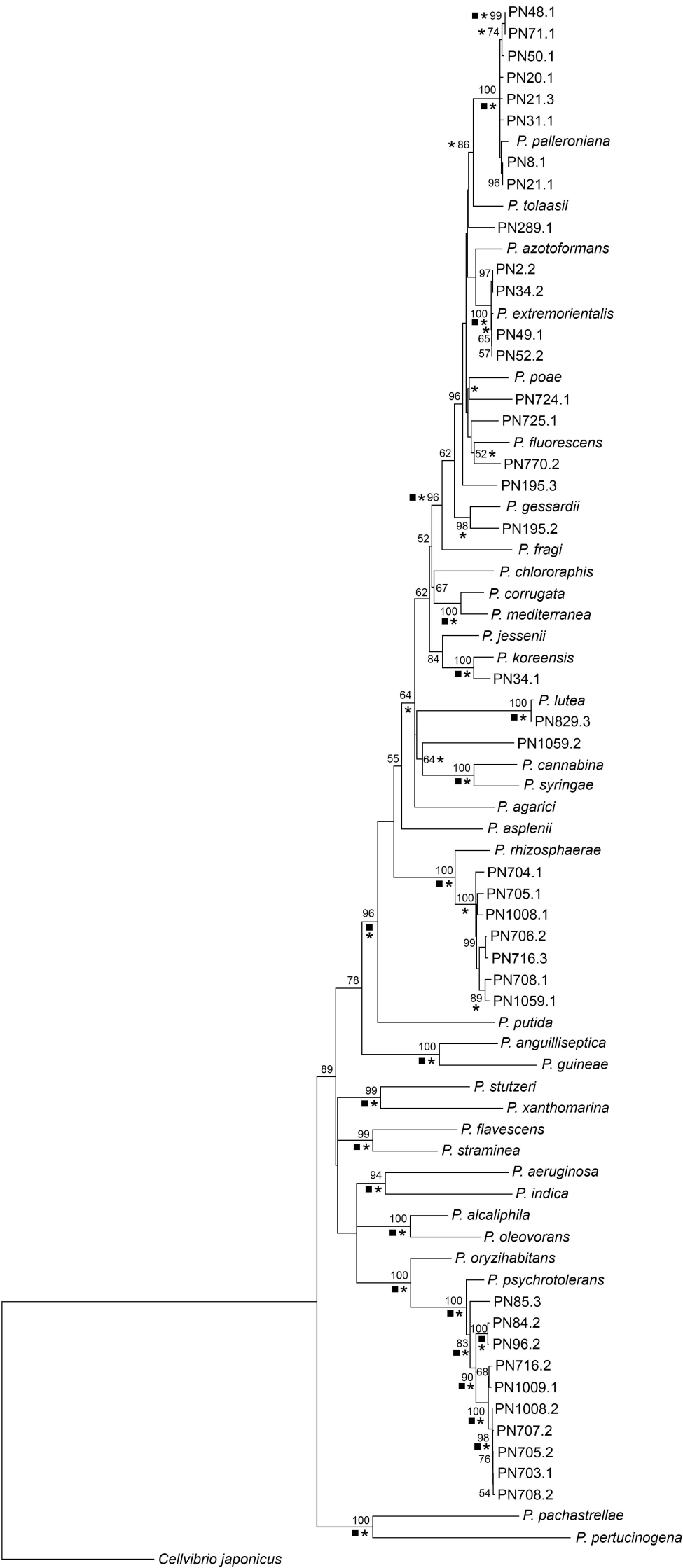

Supplement: Figure S3 — Neighbour-joining (NJ) consensus tree, based on concatenated (rrs + gyrB + rpoB + rpoD) sequences, showing the relationships of nectar-inhabiting isolates and reference (type) strains of Pseudomonas ‘sensu stricto’ species. Evolutionary distances were computed using the Jukes-Cantor method and are in the units of number of nucleotide substitutions per site. There were a total of 2601 positions in the final dataset; all positions containing gaps and missing data were eliminated. Node support values (NJ bootstrap percentages, 1000 replicates) ≥50% are shown next to the branches. Clades supported by the Maximum Likelihood (ML, ≥90% bootstrap) and Bayesian Inference (BI, ≥0.9 posterior probability) methods are indicated by filled squares and asterisks, respectively. MrBayes and PhyML settings were as explained in the main text, but using a symmetrical model of sequence evolution with gamma-distributed rate variation across sites and a proportion of invariable sites (SYM+G+I) for phylogenetic inference by the BI method. Cellvibrio japonicum Ueda107T was used as an outgroup to root the tree. GenBank accession numbers for nectar isolates are shown in Table S1, and those corresponding to reference strains in Table S3. (PDF) [file pone.0075797.s003.pdf]
