# Supplementary material for: Multilocus Sequence Analysis of Nectar Pseudomonads Reveals High Genetic Diversity and Contrasting Recombination Patterns
Source: PLoS One. 2013 Oct 8;8(10):e75797. doi: 10.1371/journal.pone.0075797 (PMC3792982; doi:10.1371/journal.pone.0075797)
Supplement: Table S1 — Details of the Pseudomonas ‘sensu stricto’ isolates characterised in this study. (PDF) [file pone.0075797.s004.pdf]

Table S1. Details of the *Pseudomonas* 'sensu stricto' isolates characterised in this study.

Isolate	Biogeographic region	Host plant (family)	GenBank accession numbers			
			rrs	*gyrB*	*rpoB*	*rpoD*
PN2.2	South Africa	*Cycnium adonense* (Orobanchaceae)	KC822762	KC822876	KC822800	KC822838
PN8.1	South Africa	*Moraea graminicola* (Iridaceae)	KC822763	KC822877	KC822801	KC822839
PN20.1	South Africa	*Ajuga ophrydis* (Lamiaceae)	KC822764	KC822878	KC822802	KC822840
PN21.1	South Africa	*Moraea graminicola* (Iridaceae)	KC822765	KC822879	KC822803	KC822841
PN21.3	South Africa	*Moraea graminicola* (Iridaceae)	KC822766	KC822880	KC822804	KC822842
PN31.1	South Africa	*Eriosema distinctum* (Fabaceae)	KC822767	KC822881	KC822805	KC822843
PN34.1	South Africa	*Ruellia cordata* (Acanthaceae)	KC822768	KC822882	KC822806	KC822844
PN34.2	South Africa	*Ruellia cordata* (Acanthaceae)	KC822769	KC822883	KC822807	KC822845
PN48.1	South Africa	*Disa crassicornis* (Orchidaceae)	KC822770	KC822884	KC822808	KC822846
PN49.1	South Africa	*Adhatoda andromeda* (Acanthaceae)	KC822771	KC822885	KC822809	KC822847
PN50.1	South Africa	*Adhatoda andromeda* (Acanthaceae)	KC822772	KC822886	KC822810	KC822848
PN52.2	South Africa	*Cycnium adonense* (Orobanchaceae)	KC822773	KC822887	KC822811	KC822849
PN71.1	South Africa	*Disa crassicornis* (Orchidaceae)	KC822774	KC822888	KC822812	KC822850
PN84.2	South Africa	*Protea welwitschii* (Proteaceae)	KC822775	KC822889	KC822813	KC822851
PN85.3	South Africa	*Protea welwitschii* (Proteaceae)	KC822776	KC822890	KC822814	KC822852
PN96.2	South Africa	*Protea welwitschii* (Proteaceae)	KC822777	KC822891	KC822815	KC822853
PN195.2	Mediterranean	*Narcissus papyraceus* (Amaryllidaceae)	KC822778	KC822892	KC822816	KC822854
PN195.3	Mediterranean	*Narcissus papyraceus* (Amaryllidaceae)	KC822779	KC822893	KC822817	KC822855
PN289.1	Mediterranean	*Fritillaria lusitanica* (Liliaceae)	KC822780	KC822894	KC822818	KC822856
PN703.1	Mediterranean	*Convolvulus althaeoides* (Convolvulaceae)	KC822781	KC822895	KC822819	KC822857
PN704.1	Mediterranean	*Convolvulus althaeoides* (Convolvulaceae)	KC822782	KC822896	KC822820	KC822858
PN705.1	Mediterranean	*Convolvulus althaeoides* (Convolvulaceae)	KC822783	KC822897	KC822821	KC822859
PN705.2	Mediterranean	*Convolvulus althaeoides* (Convolvulaceae)	KC822784	KC822898	KC822822	KC822860

PN706.2	Mediterranean	*Convolvulus althaeoides* (Convolvulaceae)	KC822785	KC822899	KC822823	KC822861
PN707.2	Mediterranean	*Fritillaria lusitanica* (Liliaceae)	KC822786	KC822900	KC822824	KC822862
PN708.1	Mediterranean	*Convolvulus althaeoides* (Convolvulaceae)	KC822787	KC822901	KC822825	KC822863
PN708.2	Mediterranean	*Convolvulus althaeoides* (Convolvulaceae)	KC822788	KC822902	KC822826	KC822864
PN716.2	Mediterranean	*Echium gaditanum* (Boraginaceae)	KC822789	KC822903	KC822827	KC822865
PN716.3	Mediterranean	*Echium gaditanum* (Boraginaceae)	KC822790	KC822904	KC822828	KC822866
PN724.1	Mediterranean	*Orobanche ramosa* (Orobanchaceae)	KC822791	KC822905	KC822829	KC822867
PN725.1	Mediterranean	*Orobanche ramosa* (Orobanchaceae)	KC822792	KC822906	KC822830	KC822868
PN770.2	Mediterranean	*Convolvulus althaeoides* (Convolvulaceae)	KC822793	KC822907	KC822831	KC822869
PN829.3	Mediterranean	*Gladiolus illyricus* (Iridaceae)	KC822794	KC822908	KC822832	KC822870
PN1008.1	Mediterranean	*Convolvulus althaeoides* (Convolvulaceae)	KC822795	KC822909	KC822833	KC822871
PN1008.2	Mediterranean	*Convolvulus althaeoides* (Convolvulaceae)	KC822796	KC822910	KC822834	KC822872
PN1009.1	Mediterranean	*Convolvulus althaeoides* (Convolvulaceae)	KC822797	KC822911	KC822835	KC822873
PN1059.1	Mediterranean	*Convolvulus althaeoides* (Convolvulaceae)	KC822798	KC822912	KC822836	KC822874
PN1059.2	Mediterranean	*Convolvulus althaeoides* (Convolvulaceae)	KC822799	KC822913	KC822837	KC822875
