# Supplementary material for: Multilocus Sequence Analysis of Nectar Pseudomonads Reveals High Genetic Diversity and Contrasting Recombination Patterns
Source: PLoS One. 2013 Oct 8;8(10):e75797. doi: 10.1371/journal.pone.0075797 (PMC3792982; doi:10.1371/journal.pone.0075797)
Supplement: Table S2 — PCR primers used in this study. (PDF) [file pone.0075797.s005.pdf]

Table S2. PCR primers used in this study.

Locus	Primer	5' → 3' sequence[a]	Ta[b]	Fragment length (bp)	Reference[d]
gyrB	UP-1E	CAGGAAACAGCTATGACCAYGSNGGNGGNAARTTYRA	62–65	966	[1]
	APrU	TGTAAAACGACGGCCAGTGCNGGRTCYTTYTCYTGRCA			
	gBMM1F[c]	GTGTCGGTKGTRAACGCCC	62–65	725	[2]
	gBMM725R[c]	GCYTCRTTSGGRTTYTCCAGCAGG			
rpoB	rpoBf1	CAGTTCATGGACCAGAACAACCCGCT	60	508	[3]
	rpoBr1	CCCATCAACGCACGGTTGGCGTC			
rpoD	PsEG30F	ATYGAAATCGCCAARCG	49–55	760	[4]
	PsEG790R	CGGTTGATKTCCTTGA			
rrs	27F	AGAGTTTGATCMTGGCTCAG	50	1465	[5]
	1492R	GGTTACCTTGTTACGACTT			

[a] Nucleotide ambiguity code: K, G or T; M, A or C; R, A or G; S, G or C; Y, C or T; N, any.

[b] Annealing temperature (°C).

[c] This set of primers was used when no amplification was achieved with UP-1E and APrU.

[d] References for this table:

[1] Yamamoto *et al.*, 2000; *Microbiology*, 146: 2385–2394.

[2] Mulet *et al.*, 2010; *Environ Microbiol*, 12: 1513–1530.

[3] Frapolli *et al.*, 2007; *Environ Microbiol*, 9: 1939–1955.

[4] Mulet *et al.*, 2009; *Mol Cell Probes*, 23: 140–147.

[5] Lane DJ, 1991. 16S/23S rRNA sequencing. *In:* Stackebrandt E, Goodfellow M (eds.) *Nucleic acid techniques in bacterial systematics*. New York: John Wiley and Sons, pp. 115–175.
