# Supplementary material for: Multilocus Sequence Analysis of Nectar Pseudomonads Reveals High Genetic Diversity and Contrasting Recombination Patterns
Source: PLoS One. 2013 Oct 8;8(10):e75797. doi: 10.1371/journal.pone.0075797 (PMC3792982; doi:10.1371/journal.pone.0075797)
Supplement: Table S3 — List of reference strains included in phylogenetic analyses. (PDF) [file pone.0075797.s006.pdf]

Table S3. List of reference strains included in phylogenetic analyses.

Species	Strain	GenBank accession numbers			
		rrs	*gyrB*	*rpoB*	*rpoD*
Cellvibrio japonicum	Ueda107^T	AF452103	NC_010995	NC_010995	NC_010995
Pseudomonas aeruginosa	DSM 50071^T	X06684	AJ633104	AJ717442	AB039607
Pseudomonas agarici	LMG 2112^T	Z76652	AB039457	AJ717477	AB039563
Pseudomonas alcaliphila	AL15-21^T	AB030583	FN554167	AJ717463	FN554448
Pseudomonas anguilliseptica	NCIMB 1949^T	X99540	FN554168	FN554726	FN554449
Pseudomonas asplenii	ATCC 23835^T	AB021397	AB039455	AJ717432	AB039593
Pseudomonas azotoformans	IAM 1603^T	D84009	AB039411	AJ717458	AB039547
Pseudomonas cannabina	CFBP 2341^T	AJ492827	FN554177	AJ717453	FN554458
Pseudomonas chlororaphis	DSM 50083^T	Z76673	D86019	AJ717478	AB039549
Pseudomonas corrugata	ATCC 29736^T	D84012	AB039460	AJ717487	AB039566
Pseudomonas extremorientalis	KMM 3447^T	AF405328	FN554182	FN554733	FN554464
Pseudomonas flavescens	B62^T	U01916	FN554183	AJ717468	FN554465
Pseudomonas fluorescens	IAM 12022^T	D84013	D86016	AJ717451	AB039545
Pseudomonas fragi	ATCC 4973^T	AF094733	FN554184	AJ717444	FN554466
Pseudomonas gessardii	CIP 105469^T	AF074384	FN554186	AJ717438	FN554468
Pseudomonas guineae	LMG 24016^T	AM491810	FN554189	FN554734	FN554471
Pseudomonas indica	IMT37^T	AF302795	FN554190	AJ717481	FN554472
Pseudomonas jessenii	CIP 105274^T	AF068259	FN554191	AJ717447	FN554473
Pseudomonas koreensis	KACC 10848^T	AF468452	FN554194	FN554737	FN554476
Pseudomonas lutea	OK2^T	AY364537	FN554198	FN554738	FN554480
Pseudomonas mediterranea	CFBP 5447^T	AF386080	AM084678	AJ717449	AM084337
Pseudomonas oleovorans	IAM 1508^T	D84018	AB039396	AJ717461	AB039601
Pseudomonas oryzihabitans	IAM 1568^T	D84004	FN554210	AJ717470	FN554494
Pseudomonas pachastrellae	KMM 330^T	AB125366	FN554212	FN554746	FN554496
Pseudomonas palleroniana	CFBP 4389^T	AY091527	FN554213	FN554747	FN554497
Pseudomonas pertucinogena	IFO 14163^T	AB021380	DQ350613	AJ717441	FN554502
Pseudomonas poae	DSM 14936^T	AJ492829	FN554219	FN554751	FN554504
Pseudomonas psychrotolerans	C36^T	AJ575816	FN554222	FN554753	FN554507
Pseudomonas putida	IAM 1236^T	D84020	AB039451	AJ717474	AB039587
Pseudomonas rhizosphaerae	IH5^T	AY152673	FN554224	FN554755	FN554510
Pseudomonas straminea	IAM 1598^T	D84023	AB039410	FN554758	AB039600
Pseudomonas stutzeri	ATCC 17588^T	AF094748	AJ617557	AJ279962	AB039618
Pseudomonas syringae	NCPPB 281^T	DQ318866	AB039428	FN554759	AB039516
Pseudomonas tolaasii	LMG 2342^T	AF255336	FN645137	AJ717467	AB039561
Pseudomonas xanthomarina	KMM 1447^T	AB176954	AM905836	FN554765	AM905872
