# Supplementary material for: Multilocus Sequence Analysis of Nectar Pseudomonads Reveals High Genetic Diversity and Contrasting Recombination Patterns
Source: PLoS One. 2013 Oct 8;8(10):e75797. doi: 10.1371/journal.pone.0075797 (PMC3792982; doi:10.1371/journal.pone.0075797)
Supplement: Table S4 — List of pollinator types for the plant species sampled. (PDF) [file pone.0075797.s007.pdf]

Table S4. List of pollinator types for the plant species sampled.

Plant species	Pollinators (insect order)
Adhatoda andromeda	Bees (Hymenoptera)
Ajuga ophrydis	Bees (Hymenoptera)
Convolvulus althaeoides	Ants and bees (Hymenoptera), beetles (Coleoptera) and flies (Diptera)
Cycnium adonense	Hawkmoths (Lepidoptera)
Disa crassicornis	Hawkmoths (Lepidoptera)
Echium gaditanum	Bees (Hymenoptera)
Eriosema distinctum	Bees and wasps (Hymenoptera), butterflies (Lepidoptera) and flies (Diptera)
Fritillaria lusitanica	Beetles (Coleoptera) and flies (Diptera)
Gladiolus illyricus	Bees (Hymenoptera) and flies (Diptera)
Moraea graminicola	Bees (Hymenoptera)
Narcissus papyraceus	Bees (Hymenoptera), butterflies (Lepidoptera) and flies (Diptera)
Orobanche ramosa	Ants(Hymenoptera)
Protea welwitschii	Bees (Hymenoptera), beetles (Coleoptera) and flies (Diptera)
Ruellia cordata	Bees (Hymenoptera)
