# Supplementary material for: Multilocus Sequence Analysis of Nectar Pseudomonads Reveals High Genetic Diversity and Contrasting Recombination Patterns
Source: PLoS One. 2013 Oct 8;8(10):e75797. doi: 10.1371/journal.pone.0075797 (PMC3792982; doi:10.1371/journal.pone.0075797)
Supplement: Table S5 — Analysis of the studied loci for the nectar groups (NGs) of pseudomonads identified in phylogenetic analyses. (PDF) [file pone.0075797.s008.pdf]

Table S5. Analysis of the studied loci for the nectar groups (NGs) of pseudomonads identified in phylogenetic analyses.

Nectar group (*n*)	Locus	Number of haplotypes	% Polymorphic sites[a]	π[b]	%G+C	dN/dS[c]	Tajima's D[d]
NG 1 (10)	*gyrB*	5	7.68	0.026	61.9	0.103	−0.449
	rpoB	5	4.34	0.017	63.1	0.017	0.500
	rpoD	8	5.90	0.018	66.1	0.402	−0.810
	rrs	2	0.14	< 0.001	53.5	NA	−1.401
NG 2 (21)	*gyrB*	18	27.07	0.079	55.2	0.090	−0.909
	rpoB	16	26.83	0.067	58.3	0.032	−1.444
	rpoD	17	36.91	0.104	60.9	0.351	−1.250
	rrs	11	3.92	0.010	53.4	NA	−0.792
NG 2' (18)	*gyrB*	15	18.38	0.060	55.2	0.095	−0.332
	rpoB	13	14.09	0.041	58.4	0.021	−0.533
	rpoD	14	24.18	0.069	60.8	0.369	−1.016
	rrs	8	1.88	0.006	53.2	NA	−0.326
NG 3 (7)	*gyrB*	6	5.06	0.024	54.7	0.000	0.284
	rpoB	6	2.71	0.010	59.8	0.052	−0.608
	rpoD	6	2.70	0.011	64.6	0.109	−0.183
	rrs	2	0.07	< 0.001	53.9	NA	−1.006

[a] Excluding gaps and missing data.

[b] Nucleotide diversity (average number of nucleotide differences per site between two sequences; [Nei M., 1987. *Molecular Evolutionary Genetics*. New York: Columbia University Press]), after Jukes and Cantor correction.

[c] Ratio between the numbers of non-synonymous and synonymous substitutions (dN and dS, respectively). NA, not applicable.

[d] Tajima's D neutrality test [Tajima, 1989; *Genetics*, 123: 585–595]. No significant ($p > 0.05$) deviation from zero was observed in any case.
