# Supplementary material for: Multilocus Sequence Analysis of Nectar Pseudomonads Reveals High Genetic Diversity and Contrasting Recombination Patterns
Source: PLoS One. 2013 Oct 8;8(10):e75797. doi: 10.1371/journal.pone.0075797 (PMC3792982; doi:10.1371/journal.pone.0075797)
Supplement: Table S6 — Recombination and mutation indices of the studied loci. (PDF) [file pone.0075797.s009.pdf]

Table S6. Recombination and mutation indices of the studied loci.[a]

Gene	No. segregating sites	ρ^{b} (×10^{-2})	θ_w^{c} (×10^{-2})	ρ/θ_w^{d}
gyrB	132	4.297 (3.442 – 5.233)	6.888	0.624
rpoB	78	3.439 (2.947 – 3.964)	5.575	0.617
rpoD	173	1.929 (1.138 – 2.950)	8.328	0.232
rrs	90	0.795 (0.278 – 1.437)	2.085	0.381
Concatenated	472	0.803 (0.681 – 0.932)	4.221	0.190

[a] Values for ρ and θ_w were calculated using LDhat [McVean *et al.*, 2002; *Genetics*, 160: 1231–1241] and are expressed per site.

[b] Rho per site (lower–upper bound, 95th percentiles).

[c] Theta per site (Watterson estimator).

[d] Rho/theta per site.
