# Supplementary material for: Multilocus Sequence Analysis of Nectar Pseudomonads Reveals High Genetic Diversity and Contrasting Recombination Patterns
Source: PLoS One. 2013 Oct 8;8(10):e75797. doi: 10.1371/journal.pone.0075797 (PMC3792982; doi:10.1371/journal.pone.0075797)
Supplement: Table S7 — Nearest neighbours on the basis of 16S rRNA (rrs) gene sequences among validly named bacterial species of all nectar strains characterised in this study, as obtained through the EzTaxon-e server. (PDF) [file pone.0075797.s010.pdf]

Table S7. Nearest neighbours on the basis of 16S rRNA (*rrs*) gene sequences among validly named bacterial species of all nectar strains characterised in this study, as obtained through the EzTaxon-e server.[a]

Nectar group	Isolate	No. of hits showing ≥ 97% similarity[b]	Best hit (accession no., similarity)	Second and third best hits (accession no., similarity)
NG 1	PN703.1	3	*Pseudomonas psychrotolerans* C36^T (AJ575816, 99.59%)	*Pseudomonas oryzihabitans* IAM 1568^T (AM262973, 99.45%)<br>*Pseudomonas oleovorans* subsp. *oleovorans* IAM 1508^T (D84018, 99.13%)
NG 1	PN716.2	3	*Pseudomonas psychrotolerans* C36^T (AJ575816, 99.59%)	*Pseudomonas oryzihabitans* IAM 1568^T (AM262973, 99.45%)<br>*Pseudomonas oleovorans* subsp. *oleovorans* IAM 1508^T (D84018, 99.13%)
NG 1	PN705.2	3	*Pseudomonas psychrotolerans* C36^T (AJ575816, 99.66%)	*Pseudomonas oryzihabitans* IAM 1568^T (AM262973, 99.52%)<br>*Pseudomonas oleovorans* subsp. *oleovorans* IAM 1508^T (D84018, 99.19%)
NG 1	PN708.2	3	*Pseudomonas psychrotolerans* C36^T (AJ575816, 99.45%)	*Pseudomonas oryzihabitans* IAM 1568^T (AM262973, 99.31%)<br>*Pseudomonas oleovorans* subsp. *oleovorans* IAM 1508^T (D84018, 98.99%)
NG 1	PN707.2	3	*Pseudomonas psychrotolerans* C36^T (AJ575816, 99.66%)	*Pseudomonas oryzihabitans* IAM 1568^T (AM262973, 99.52%)<br>*Pseudomonas oleovorans* subsp. *oleovorans* IAM 1508^T (D84018, 99.19%)
NG 1	PN85.3	3	*Pseudomonas psychrotolerans* C36^T (AJ575816, 99.66%)	*Pseudomonas oryzihabitans* IAM 1568^T (AM262973, 99.52%)<br>*Pseudomonas oleovorans* subsp. *oleovorans* IAM 1508^T (D84018, 99.19%)
NG 1	PN84.2	3	*Pseudomonas psychrotolerans* C36^T (AJ575816, 99.66%)	*Pseudomonas oryzihabitans* IAM 1568^T (AM262973, 99.52%)<br>*Pseudomonas oleovorans* subsp. *oleovorans* IAM 1508^T (D84018, 99.19%)
NG 1	PN96.2	3	*Pseudomonas psychrotolerans* C36^T (AJ575816, 99.66%)	*Pseudomonas oryzihabitans* IAM 1568^T (AM262973, 99.59%)<br>*Pseudomonas oleovorans* subsp. *oleovorans* IAM 1508^T

				(D84018, 99.26%)
NG 1	PN1008.2	3	*Pseudomonas psychrotolerans* C36^T (AJ575816, 99.66%)	*Pseudomonas oryzihabitans* IAM 1568^T (AM262973, 99.52%)<br>*Pseudomonas oleovorans* subsp. *oleovorans* IAM 1508^T (D84018, 99.19%)
NG 1	PN1009.1	3	*Pseudomonas psychrotolerans* C36^T (AJ575816, 99.66%)	*Pseudomonas oryzihabitans* IAM 1568^T (AM262973, 99.59%)<br>*Pseudomonas oleovorans* subsp. *oleovorans* IAM 1508^T (D84018, 99.19%)
NG 2	PN195.2	79	*Pseudomonas brenneri* CFML 97-391^T (AF268968, 99.52%)	*Pseudomonas migulae* CIP 105470^T (AF074383, 99.33%)<br>*Pseudomonas proteolytica* CMS 64^T (AJ537603, 99.33%)
NG 2	PN195.3	44	*Pseudomonas azotoformans* IAM1603^T (D84009, 99.66%)	*Pseudomonas libanensis* CIP 105460^T (AF057645, 99.53%)<br>*Pseudomonas gessardii* CIP 105469^T (AF074384, 99.53%)
NG 2	PN289.1	73	*Pseudomonas lurida* DSM 15835^T (AJ581999, 99.73%)	*Pseudomonas costantinii* CFBP 5705^T (AF374472, 99.66%)<br>*Pseudomonas extremaustralis* 14-3^T (AHIP01000073, 99.60%)
NG 2	PN724.1	74	*Pseudomonas extremaustralis* 14-3^T (AHIP01000073, 99.87%)	*Pseudomonas antarctica* CMS 35^T (AJ537601, 99.73%)<br>*Pseudomonas grimontii* CFML 97-514^T (AF268029, 99.73%)
NG 2	PN725.1	44	*Pseudomonas brenneri* CFML 97-391^T (AF268968, 99.66%)	*Pseudomonas trivialis* DSM 14937^T (AJ492831, 99.40%)<br>*Pseudomonas proteolytica* CMS 64^T (AJ537603, 99.39%)
NG 2	PN770.2	76	*Pseudomonas simiae* OLi^T (AF936933, 99.85%)	*Pseudomonas poae* DSM 14936^T (AJ492829, 99.80%)<br>*Pseudomonas trivialis* DSM 14937^T (AJ492831, 99.73%)
NG 2	PN829.3	55	*Pseudomonas lutea* OK2^T (AY364537, 99.87%)	*Pseudomonas graminis* DSM 11363^T (Y11150, 99.26%)<br>*Pseudomonas umsongensis* Ps 3-10^T (AF468450, 98.90%)
NG 2	PN1059.2	59	*Pseudomonas syringae* ATCC 19310^T (AJ308316, 98.68%)	*Pseudomonas chlororaphis* subsp. *chlororaphis* DSM 50083^T (Z76673, 98.51%)<br>*Pseudomonas kilonensis* 520-20^T (AJ292426, 98.39%)
NG 2	PN34.1	62	*Pseudomonas koreensis* Ps9-14^T (AF468452, 100%)	*Pseudomonas reinekei* Mt-1^T (AM293565, 99.59%)<br>*Pseudomonas jessenii* CIP 105274^T (AF068259, 99.46%)
NG 2	PN49.1	50	*Pseudomonas extremorientalis* KMM 3447^T (AF405328, 99.93%)	*Pseudomonas simiae* OLi^T (AJ936933, 99.78%)<br>*Pseudomonas poae* DSM 14936^T (AJ492829, 99.65%)

NG 2	PN52.2	51	*Pseudomonas extremorientalis* KMM 3447^T (AF405328, 100%)	*Pseudomonas simiae* OLi^T (AJ936933, 99.78%)<br>*Pseudomonas poae* DSM 14936^T (AJ492829, 99.65%)
NG 2	PN2.2	79	*Pseudomonas extremorientalis* KMM 3447^T (AF405328, 99.93%)	*Pseudomonas simiae* OLi^T (AJ936933, 99.78%)<br>*Pseudomonas poae* DSM 14936^T (AJ492829, 99.67%)
NG 2	PN34.2	52	*Pseudomonas extremorientalis* KMM 3447^T (AF405328, 100%)	*Pseudomonas simiae* OLi^T (AJ936933, 99.78%)<br>*Pseudomonas poae* DSM 14936^T (AJ492829, 99.65%)
NG 2	PN8.1	54	*Pseudomonas palleroniana* CFBP 4389^T (AY091527, 99.78%)	*Pseudomonas costantinii* CFBP 5705^T (AF374472, 99.57%)<br>*Pseudomonas lurida* DSM 15835^T (AJ581999, 99.51%)
NG 2	PN21.1	54	*Pseudomonas palleroniana* CFBP 4389^T (AY091527, 99.78%)	*Pseudomonas costantinii* CFBP 5705^T (AF374472, 99.57%)<br>*Pseudomonas lurida* DSM 15835^T (AJ581999, 99.51%)
NG 2	PN31.1	54	*Pseudomonas palleroniana* CFBP 4389^T (AY091527, 99.78%)	*Pseudomonas costantinii* CFBP 5705^T (AF374472, 99.57%)<br>*Pseudomonas lurida* DSM 15835^T (AJ581999, 99.51%)
NG 2	PN50.1	52	*Pseudomonas palleroniana* CFBP 4389^T (AY091527, 99.79%)	*Pseudomonas costantinii* CFBP 5705^T (AF374472, 99.59%)<br>*Pseudomonas lurida* DSM 15835^T (AJ581999, 99.53%)
NG 2	PN48.1	54	*Pseudomonas palleroniana* CFBP 4389^T (AY091527, 99.78%)	*Pseudomonas costantinii* CFBP 5705^T (AF374472, 99.57%)<br>*Pseudomonas lurida* DSM 15835^T (AJ581999, 99.51%)
NG 2	PN71.1	54	*Pseudomonas palleroniana* CFBP 4389^T (AY091527, 99.70%)	*Pseudomonas costantinii* CFBP 5705^T (AF374472, 99.49%)<br>*Pseudomonas lurida* DSM 15835^T (AJ581999, 99.42%)
NG 2	PN20.1	54	*Pseudomonas palleroniana* CFBP 4389^T (AY091527, 99.78%)	*Pseudomonas costantinii* CFBP 5705^T (AF374472, 99.57%)<br>*Pseudomonas lurida* DSM 15835^T (AJ581999, 99.51%)
NG 2	PN21.3	55	*Pseudomonas palleroniana* CFBP 4389^T (AY091527, 99.79%)	*Pseudomonas costantinii* CFBP 5705^T (AF374472, 99.58%)<br>*Pseudomonas lurida* DSM 15835^T (AJ581999, 99.52%)
NG 3	PN704.1	53	*Pseudomonas rhizosphaerae* IH5^T (AY152673, 99.60%)	*Pseudomonas graminis* DSM 11363^T (Y11150, 98.72%)<br>*Pseudomonas lutea* OK2^T (AY364537, 98.59%)
NG 3	PN716.3	50	*Pseudomonas rhizosphaerae* IH5^T (AY152673, 99.66%)	*Pseudomonas graminis* DSM 11363^T (Y11150, 98.86%)<br>*Pseudomonas lutea* OK2^T (AY364537, 98.73%)
NG 3	PN705.1	49	*Pseudomonas rhizosphaerae* IH5^T (AY152673, 99.67%)	*Pseudomonas graminis* DSM 11363^T (Y11150, 98.80%)

				Pseudomonas lutea OK2^T (AY364537, 98.60%)
NG 3	PN706.2	51	*Pseudomonas rhizosphaerae* IH5^T (AY152673, 99.60%)	*Pseudomonas graminis* DSM 11363^T (Y11150, 98.73%)<br>*Pseudomonas lutea* OK2^T (AY364537, 98.59%)
NG 3	PN708.1	57	*Pseudomonas rhizosphaerae* IH5^T (AY152673, 99.66%)	*Pseudomonas graminis* DSM 11363^T (Y11150, 98.79%)<br>*Pseudomonas lutea* OK2^T (AY364537, 98.66%)
NG 3	PN1008.1	57	*Pseudomonas rhizosphaerae* IH5^T (AY152673, 99.66%)	*Pseudomonas graminis* DSM 11363^T (Y11150, 98.86%)<br>*Pseudomonas lutea* OK2^T (AY364537, 98.73%)
NG 3	PN1059.1	59	*Pseudomonas rhizosphaerae* IH5^T (AY152673, 99.60%)	*Pseudomonas graminis* DSM 11363^T (Y11150, 98.73%)<br>*Pseudomonas lutea* OK2^T (AY364537, 98.59%)

[a] http://eztaxon-e.ezbiocloud.net/ (Kim *et al.*, 2012; *Int J Syst Evol Microbiol*, 62: 716–721) [last accessed 28 Mar. 2013].

[b] Number of reference sequences in the database showing ≥ 97% DNA similarity with the query sequence.
