# Supplementary material for: Multilocus Sequence Analysis of Nectar Pseudomonads Reveals High Genetic Diversity and Contrasting Recombination Patterns
Source: PLoS One. 2013 Oct 8;8(10):e75797. doi: 10.1371/journal.pone.0075797 (PMC3792982; doi:10.1371/journal.pone.0075797)
Supplement: Table S8 — Tentative classification of the nectar-inhabiting Pseudomonas characterised in this study. (PDF) [file pone.0075797.s011.pdf]

**Table S8.** Tentative classification of the nectar-inhabiting *Pseudomonas* characterised in this study.

| Taxonomic lineage, group and/or subgroup[a] (nectar group(s)) | Isolates |
| --- | --- |
| *P. oryzihabitans* group (NG 1) | PN84.2, PN85.3, PN96.2, PN703.1, PN705.2, PN707.2, PN708.2, PN716.2, PN1008.2, PN1009.1 |
| *P. fluorescens* lineage | |
| *P. fluorescens* group | |
| *P. fluorescens* subgroup (NG 2') | PN2.2, PN8.1, PN20.1, PN21.1, PN21.3, PN31.1, PN34.2, PN48.1, PN49.1, PN50.1, PN52.2, PN71.1, PN195.3, PN289.1, PN724.1, PN725.1, PN770.2 |
| *P. gessardii* subgroup (NG 2') | PN195.2 |
| *P. koreensis* subgroup (NG 2) | PN34.1 |
| *P. lutea* group (NG 2) | PN829.3 |
| *P. syringae* group (NG 2) | PN1059.2 |
| *P. rhizosphaerae* 'group' (NG 3) | PN704.1, PN705.1, PN706.2, PN708.1, PN716.3, PN1008.1, PN1059.1 |

[a] According to Mulet *et al.* [2010; *Environ Microbiol*, 12: 1513–1530].
